# A miRNA Signature of Prion Induced Neurodegeneration

**DOI:** 10.1371/journal.pone.0003652

**Published:** 2008-11-06

**Authors:** Reuben Saba, Chelsey D. Goodman, Rhiannon L. C. H. Huzarewich, Catherine Robertson, Stephanie A. Booth

**Affiliations:** 1 Molecular PathoBiology, National Microbiology Laboratory, Canadian Science Center for Human and Animal Health, Public Health Agency of Canada, Winnipeg, Canada; 2 Department of Medical Microbiology and Infectious Diseases, Faculty of Medicine, University of Manitoba, Winnipeg, Canada; University of Massachusetts Medical School, United States of America

## Abstract

MicroRNAs (miRNAs) are small, non-coding RNA molecules which are emerging as key regulators of numerous cellular processes. Compelling evidence links miRNAs to the control of neuronal development and differentiation, however, little is known about their role in neurodegeneration. We used microarrays and RT-PCR to profile miRNA expression changes in the brains of mice infected with mouse-adapted scrapie. We determined 15 miRNAs were de-regulated during the disease processes; miR-342-3p, miR-320, let-7b, miR-328, miR-128, miR-139-5p and miR-146a were over 2.5 fold up-regulated and miR-338-3p and miR-337-3p over 2.5 fold down-regulated. Only one of these miRNAs, miR-128, has previously been shown to be de-regulated in neurodegenerative disease. De-regulation of a unique subset of miRNAs suggests a conserved, disease-specific pattern of differentially expressed miRNAs is associated with prion–induced neurodegeneration. Computational analysis predicted numerous potential gene targets of these miRNAs, including 119 genes previously determined to be also de-regulated in mouse scrapie. We used a co-ordinated approach to integrate miRNA and mRNA profiling, bioinformatic predictions and biochemical validation to determine miRNA regulated processes and genes potentially involved in disease progression. In particular, a correlation between miRNA expression and putative gene targets involved in intracellular protein-degradation pathways and signaling pathways related to cell death, synapse function and neurogenesis was identified.

## Introduction

Transmissible spongiform encephalopathies (TSEs), or prion diseases, are an invariably fatal class of neurodegenerative disorders. Substantial evidence links the development of disease with the conformational conversion of PrP^C^, a normal cellular protein, into PrP^Sc^, a structural isoform that is infectious [1]. In contrast, very little is known about the molecular pathways triggered by these agents that lead to the damage, and ultimate death of neurons. Molecular events leading to prion induced neurodegeneration are poorly characterized and this is an important field of ongoing research, potentially leading to the development of treatments. Efforts have been made to identify the genes involved in pathogenesis at various stages of the disease in both *in vivo* and *in vitro* models by microarray analysis of brain tissue [2]–[6]. Specific *in vitro* models have looked at the involvement of genes expressed in a number of different CNS cell types including neuronal cells [7]–[9], microglial cells [10], [11] and astrocytes [12]. While it is useful to determine all those genes that are potentially involved in the disease process it is especially important for us to identify the key regulators of these processes; especially the pathways that lead to death in neurons. These may include transcription factors and regulatory protein kinases.

The recent identification of microRNAs (miRNAs) has revealed an important layer of post-transcriptional gene regulation. MiRNAs are a class of small (∼18–25 nucleotides long), endogenous, non-coding RNA molecules that post-transcriptionally regulate protein coding mRNA in both plants and animals. Several hundred miRNAs have been identified, many of which are tissue-specific and/or temporally regulated in their expression [13]. The biological function of only a small fraction of these miRNAs have been reported in detail and point to their involvement in a variety of developmental and physiological processes. MiRNAs mediate gene silencing by binding to specific target sites within the 3′UTR of mRNA to either block translation or to bring about degradation of transcripts. These effects can be subtle and in a number of instances result in ‘fine-tuning’ of gene expression [14], [15]. Among vertebrate miRNAs, where the complementarity between the miRNA and its target site on the mRNA is imperfect, there exists the possibility for each miRNA to regulate hundreds of potential targets. Unexpectedly, recent work has shown miRNAs to be involved in the activation of gene expression in certain conditions such as cellular stress [16], [17]. Aberrant miRNA expression patterns have been described in several diseases including neurodegenerative diseases [18]–[21]. Furthermore, using Purkinje cells as a model system to analyze the role of miRNAs in postmitotic, differentiated neurons, the inactivation of Dicer enzyme leads to rapid loss of cell-specific miRNAs and the slow degeneration of the Purkinje cells leading to ataxia and severe cerebellar dysfunction, suggesting miRNAs are required to maintain neuronal integrity [22].

Here we present the first comprehensive analysis of miRNA expression in the brain during prion-induced neurodegeneration. We report the differential expression of 15 miRNAs, the majority of which are up-regulated during prion disease. Using miRNA target prediction programs in combination with mRNA profiling we have identified pathways and networks of genes in which miRNA regulation is potentially important during mouse scrapie.

## Results

### De-regulation of miRNA expression during prion disease in mice

To determine whether the expression profile of miRNAs changes during prion disease, we analyzed the global expression of mature miRNAs in the brains of mice intra-cerebrally inoculated with scrapie strain 22A (n = 6). We used a custom, in-house manufactured miRNA microarray platform to profile the expression of miRNAs [23]. In total, we determined differential expression of 14 miRNAs in the brains of scrapie infected versus mock-infected mice using this microarray. In some cases, the differential signal was identified by multiple probes on the array which were designed to identify species specific miRNA sequences, some of which differ by one or two nucleotides from the mouse sequence. We used qRT-PCR (TaqMan® miRNA Assays) to validate the expression of these 14 miRNAs, 8 up-regulated and 6 down-regulated. Our criteria for TaqMan® validation was that at least 5 of the 6 infected mice showed a mean fold change >1.75. Using this criteria we were able to confirm the expression levels of 7/8 up-regulated miRNAs and 1/6 down-regulated miRNAs, shown in Table 1.

**Table 1:** MiRNAs identified as differentially expressed in CNS tissues of scrapie infected mice.

	Average fold Change by TaqMan® miRNA Assay	Identified by miRNA Microarray Probes			miRNA abundance in brain tissues (qRT-PCR)*
		Mouse	Human	Rat	
**Up-regulated**					
miR-342-3p	3.3	**•**	**•**	**•**	**+++**
miR-320	2.9		**•**	**•**	**++**
miR-let-7b	2.7	**•**	**•**	**•**	**++++**
miR-328	2.7	**•**	**•**	**•**	**+++**
miR-191	2.0	**•**	**•**	**•**	**+++**
miR-let-7d	1.9	**•**	**•**	**•**	**++++**
miR-370	1.9	**•**	**•**		**+**
miR-128	3.0				**++**
miR-139-5p	3.0				**+**
miR-146a	2.6				**++**
miR-339-5p	2.3				**+**
miR-203	2.1				**+**
miR-181a-1*	1.9				**+**
**Down-regulated**					
miR-338-3p	−6.5	**•**	**•**	**•**	**++**
miR-337-3p	−3.1				**+**

MiRNAs which exhibit a trend towards up-regulation; miR-200a, miR-200b, miR-26a, miR-186, miR-331-3p, miR-152, miR-221.

* Results from [23].

We have previously found that TaqMan® miRNA Assays are more sensitive for detection of low abundance miRNAs than our custom microarray [23]. We therefore screened a further 157 miRNAs for differential expression using the TaqMan® panel. We determined that 114 of these miRNAs were present at detectable levels in mouse brain and that a further 6 up-regulated miRNAs and 1 down-regulated miRNA that adhered to our criteria (Data S1). These disease-associated miRNAs are also listed in Table 1. In addition, we detected a trend in the up-regulation of the following miRNAs, although the values were not consistent enough to meet the criteria that 5 of the 6 infected mice show a fold change >1.75; miR-200a, miR-200b, miR-26a, miR-186, miR-331-3p, miR-152, miR-221. Included as annotation in Table 1 is the relative abundance of each miRNA in normal CNS tissues, as measured by qRT-PCR [23]. The de-regulated miRNAs exhibit a full range of expression levels in normal CNS tissue from miRNAs found at very low abundance in the brain, such as miR-203 which is preferentially expressed in endothelial cells, to highly expressed miRNAs such as let-7b and let-7d.

### Computational predictions of the putative targets of de-regulated miRNAs

**Figure 1:**
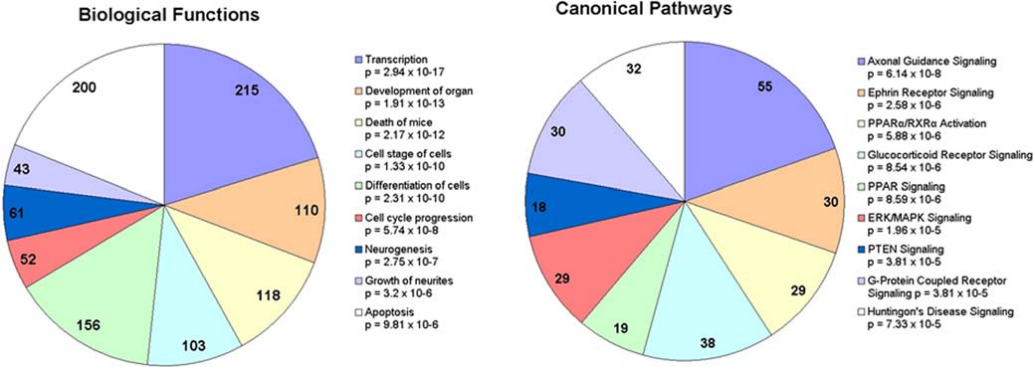
Classification of prion disease-specific miRNA target genes into, A, functional groups and B, canonical pathways.

**Figure 2:**
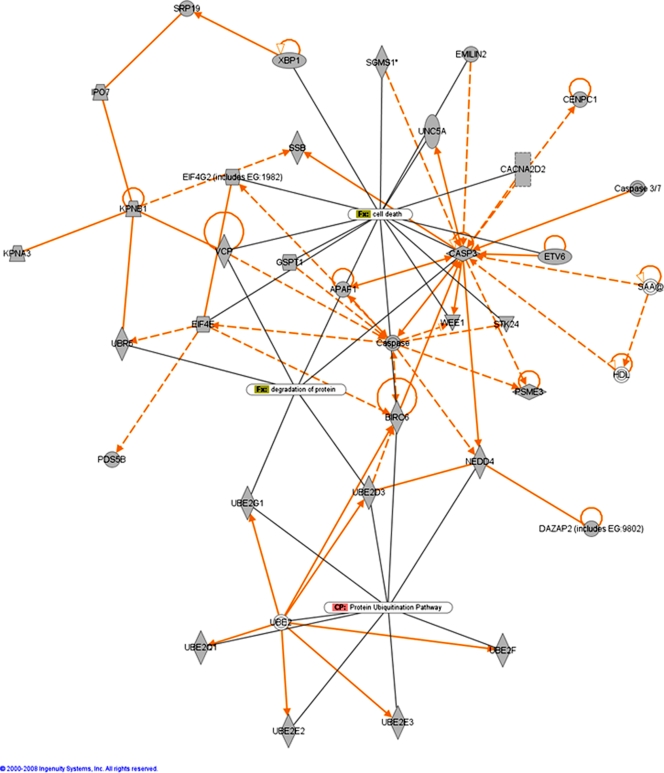
Functional relationships of miRNA target genes involved in the ubiquitin-proteasome pathway and unfolded-protein response and cell death responses. This diagram shows the direct (solid lines) and indirect (dashed lines) interactions reported for these putative target genes (grey shading) of miRNAs de-regulated in scrapie infection in mice. Biological network analysis was performed using Ingenuity Pathway Analysis (IPA).

A single vertebrate miRNA can target hundreds of mRNA transcripts for either translation repression or degradation [24], [25]. We used the computational algorithms miRanda, PicTar, and TargetScan to identify prion-related potential target genes of the de-regulated miRNAs based on seed-sequence homology with 3′ UTRs of mRNA transcripts [26]–[28]. We relied on a consensus-based approach by considering only those target genes that were similarly predicted by two or more of these algorithms. The total number of target genes predicted for the differentially expressed miRNAs by two or more of the leading target finding algorithms was 1282, which are listed in Data S2. Of these, 753 are annotated in the Ingenuity Pathway Analysis (IPA) database in terms of their biological function. The most enriched functional groups of genes amongst the miRNA targets, and the canonical pathways within which they play a role, are provided in Figure 1. Putative target genes include many previously reported to play a role in various neurodegenerative disorders, especially those linked with the accumulation of aggregated proteins. MiRNAs have already been shown to play a role, possibly positively influencing neuronal survival, in the modulation of Ataxin-3 (ATXN3) in the polyglutamine disease spinocerebellar ataxia type 3 (SCA3) where the miRNA *ban* was determined to act down-stream of ATXN-3 toxicity [18]. Bioinformatic analysis identified Ataxin-1 (ATXN1) itself as a potential target of miRNAs up-regulated in this study. The disruption in the clearance of protein aggregates by the ubiquitin-proteasome (UPS) pathway has also been found to play a role in pathogenesis of several neurodegenerative conditions including late-onset Parkinson's and Huntington's disease [29]. Over 30 genes involved in the UPS pathway are potential targets of the de-regulated miRNAs we have identified. These include ubiquitin, ubiquitin-conjugating enzymes and the ubiquitin protein ligase NEDD4; also amongst the targets are genes involved in the unfolded-protein response, valosin-containing protein, VCP (CDC48/p97), which is important for the shuttling of ubiquitinated proteins from the endoplasmic reticulum (ER) to the proteasome, and the transcription factor X-box binding protein 1, XBP1, activated following the accumulation of unfolded proteins in the ER. A biological network generated by the Ingenuity Pathway Analysis tool (IPA) is provided in Figure 2 which shows the high degree of interactions between the potential targets of selected genes that are involved in the UPS and protein degradation pathways that are linked to cell death.

**Figure 3:**
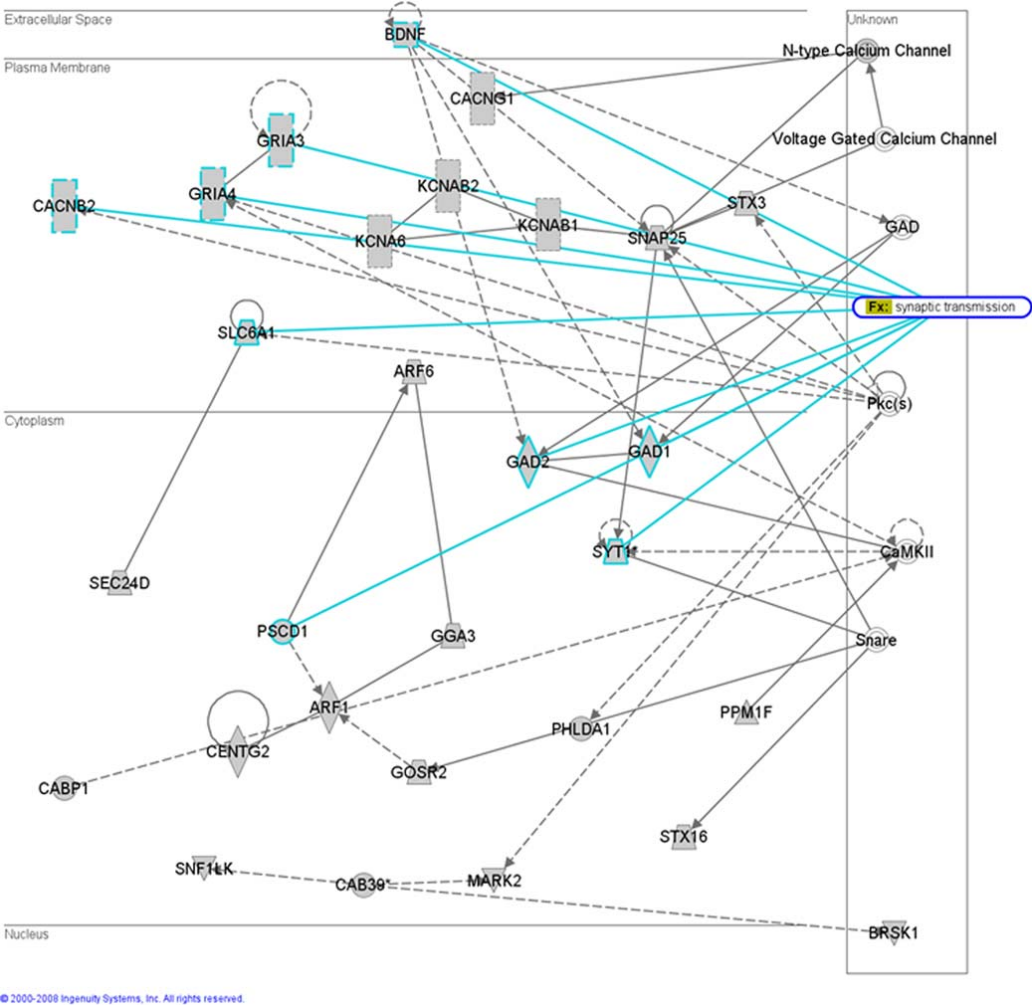
A network of 29 miRNA target genes that have a high probability of interacting within neuronal synapses.

The UPS is also known to play a role in the normal functioning of synapses and defective proteolysis could cause the synaptic dysfunction observed early in prion diseases [30]–[33]. Other miRNA gene targets are also involved in the modulation of synapse function including the neurotrophic factors, brain derived neurotrophic factor (BDNF) and neurotrophin 3 (NT3) which have crucial roles in the modulation of synaptic plasticity and in maintenance of survival of neuronal populations [34], [35]. Figure 3 shows an IPA generated network of 29 miRNA target genes that have a high probability of interacting within neuronal synapses. Genes coding for other factors involved in synapse function including neurotransmitter receptors, gamma-aminobutyric acid (GABA), glutamate receptors (GABRA1, GABRB2, GABRB3, GRIA3, GRIA4) and SNAP25 are also potential targets of the up-regulated miRNAs from the study. Expression of SNAP25 has recently been shown to be down-regulated by miR-128 leading to perturbation of neuronal activity [36]. We have previously shown SNAP25 to be down regulated in scrapie infected mice, potentially targeted by the up-regulated miR-128.

### Enrichment of regulatory motifs in miRNA targets

**Figure 4:**
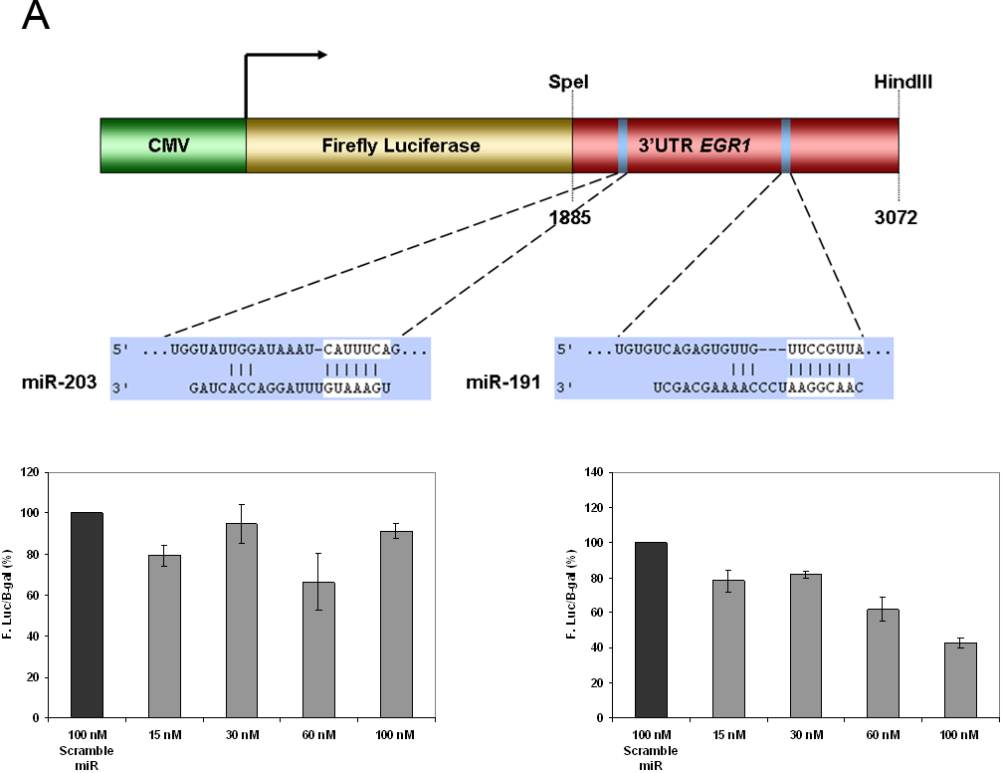
MiRNA target gene validation of EGR1 using a luciferase reporter gene assay. A, Schematic representation of luciferase constructs used for reporter assays. Specifically, EGR1 3′UTR construct in pMIR-REPORT for transfection into HeLa cells. B, Dose dependent reduction in the expression of luciferase activity when HeLa cells are co-transfected with miR-203 and C, co-transfection with miR-191.

Examination of the promoter regions of putative miRNA target genes using the FatiGO tool revealed that regulatory motifs for a small number of transcription factors including, E2F-1 (p = 6.01×10^−14^), KROX (p = 9.34×10^−14^), MAZ (p = 2.23×10^−11^) and PAX6 (p = 1.76×10^−9^) are significantly enriched in comparison to genes in the mouse genome as a whole [37]. This suggests that the de-regulated miRNAs are involved in co-ordinate regulation of networks of functionally related genes. We have previously found the KROX family transcription factor EGR1 to be differentially expressed in microarray studies of RNA extracted from whole scrapie-infected brains [4]. MAZ is an inflammation-responsive transcription factor that has been co-localized to pathologic structures in Alzheimer's disease brain while up regulation of E2F1 has been shown to trigger cell cycle re-entry of post-mitotic neurons leading to the initiation of apoptosis in neurodegeneration associated with Parkinson's disease [38], [39]. PAX6 has an important but complex role in neurogenesis that is dependent on its expression level influencing cell proliferation, differentiation and apoptosis [40]–[43]. EGR1 is itself a putative target of two miRNAs identified in this study, miR-203 and miR-191. We used a luciferase reporter assay to determine that miR-191, but not miR-203, is a potential regulator of EGR1, Figure 4.

### Comparative analysis of networks

**Figure 5:**
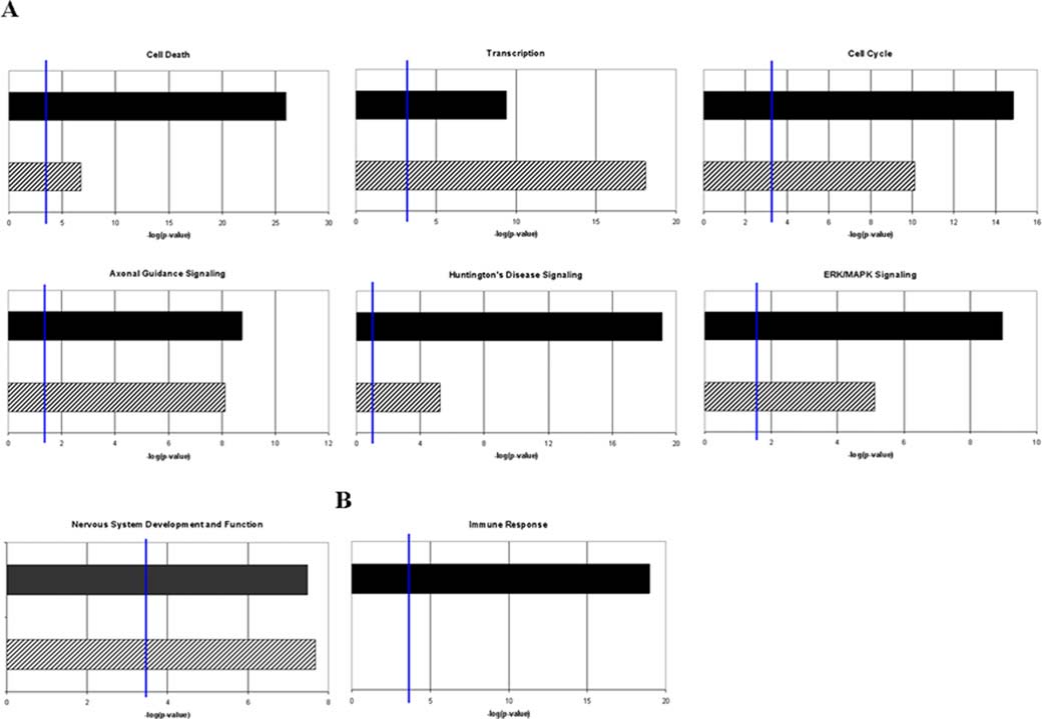
Comparison of the GO assignments for putative target genes of differentially expressed miRNAs with genes identified to be differentially regulated in prion diseases. A, De-regulated target genes that show a correlation with de-regulated prion related genes. B, Functionally-related genes that are strongly de-regulated in mouse scrapie but have little or no representation among putative target genes of miRNAs that exhibit differential expression in prion disease (solid bars, genes de-regulated in mouse scrapie, hatched bars, putative targets of de-regulated miRNAs).

**Figure 6:**
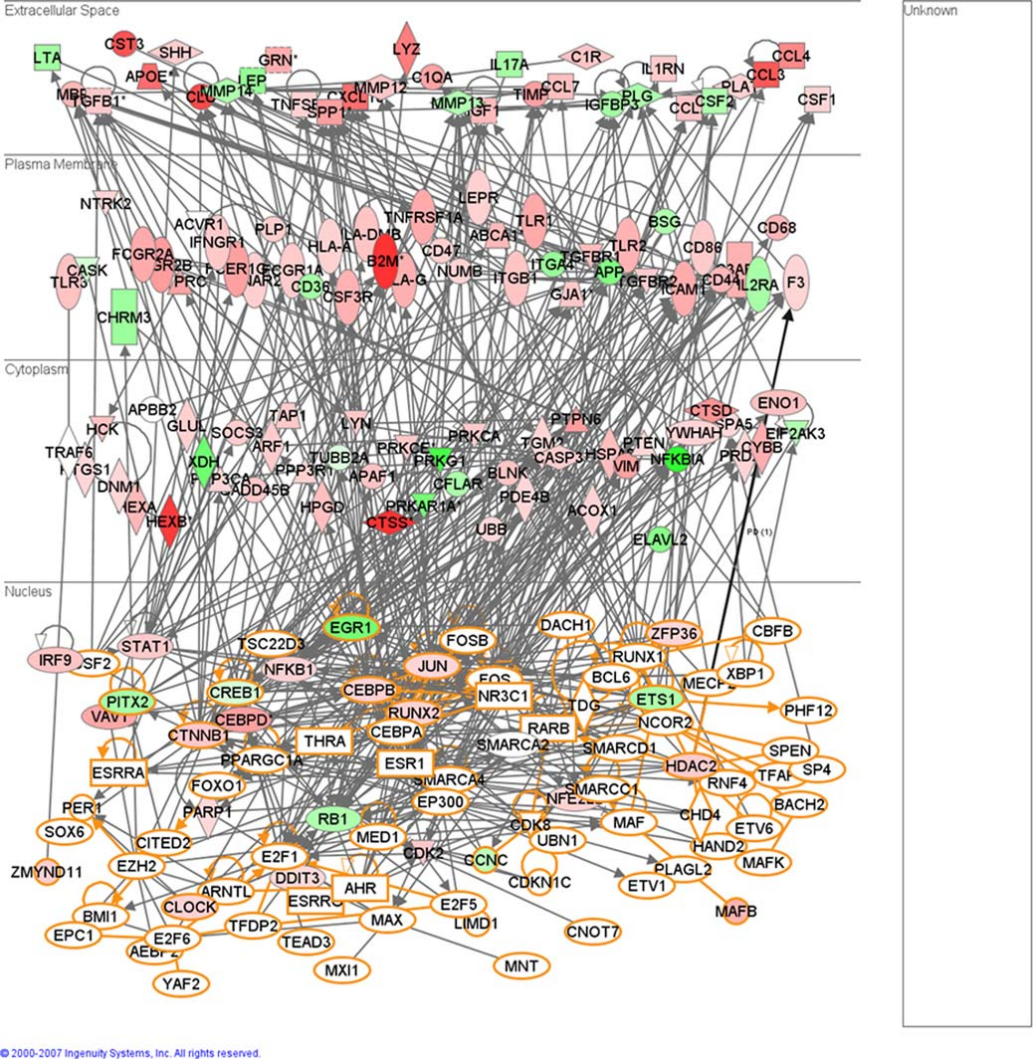
Functional relationships of de-regulated immune response-related genes in the brains of mice infected with scrapie and the putative transcription regulator genes that are targets of miRNAs similarly de-regulated. This biological network illustrates the potential for indirect regulation of immune-response related genes by miRNAs via modulation of transcriptional regulators. Nodes colored in red denote up-regulated genes in mouse scrapie whereas green denotes down-regulated genes. Nodes with an orange border denote the putative targets of miRNAs deregulated in mouse scrapie.

The overlap between putative targets of miRNAs and the expression of mRNAs provides information on the biological functions and networks of genes regulated by specific miRNAs. We used the IPA tool to identify linkages between putative targets of de-regulated miRNAs and differentially expressed genes in mouse scrapie [44], [45]. The genes used in the comparison comprised 349 prion related genes (PRGs) identified in a previous study using in-house constructed microarrays consisting of cDNAs, plus a further 684 genes identified using Agilent whole genome mouse arrays [46]. In total, 119 (12%) of these genes are predicted targets of de-regulated miRNAs by 2 or more miRNA target prediction algorithms. This may be an inverse relationship, for example, over-expressed miRNAs and down-regulated target genes or down-regulated miRNAs and over-expressed target genes, however as miRNAs may function indirectly in many instances, or indeed as activators of expression, a general correlation in functionality of the two groups is of interest [14], [15]. The results of this analysis in terms of biological processes and pathways into which miRNA targets and de-regulated genes are correlated are provided in Figure 5. The closest linkages between the data sets were found to be in the significant enrichment for genes involved in cell death, regulation of the cell cycle, nervous system development and function and cell signaling pathways. Interestingly, the largest groups of de-regulated genes in RNA from brains affected by prion disease are the inflammatory response genes and these were not significantly represented amongst the putative targets of the de-regulated miRNAs. MiRNAs may be indirect regulators of these genes at the level of transcription by regulation of transcription factors, given that approximately a quarter of the putative targets of de-regulated miRNAs are involved in this process. We determined that many of these miRNA transcription related targets are potentially directly involved in the expression of the 164 inflammation-related genes de-regulated in mouse scrapie; a gene interaction network to illustrate this is provided in Figure 6. These data suggest that aberrant expression of miRNAs may modulate the activity of particular transcription factors and alter gene regulatory circuits at multiple hierarchical levels leading to the disease phenotype.

## Discussion

This is the first report that comprehensively analyzes the brain miRNA expression profile during prion-induced neurodegeneration in mouse scrapie. Only a small number of miRNAs have so far been associated with neurodegenerative processes: miR-133b, miR-9, miR-125b, miR-132, miR-124a, miR-219 and miR-128 [19], [20], [47]. Of these, only miR-128 was significantly up-regulated in the brains of scrapie infected mice. This miRNA has previously been reported as increased in Alzheimer's hippocampus [19]. Interestingly, we noted significant up-regulation of miR-342-3p which is also one of three de-regulated miRNAs independently identified in the brains of BSE-infected macaques (Dirk Motzkus, German Primate Center, personal communication). A second miRNA identified in this study, miR-26a, exhibited a trend towards up-regulation in our study (>1.3 fold up-regulated in 5 out of 6 mice). The fact that we detected de-regulation of a unique subset of miRNAs, but which are similarly expressed in different models of TSEs, suggests a conserved, disease-specific pattern of differentially expressed miRNAs is associated with prion–induced neurodegeneration.

Recent studies in Dicer-deficient mice have highlighted the importance of miRNAs in regulating neuronal function. In recent studies the loss of Dicer in postmitotic Purkinje neurons and midbrain dopamine neurons resulted in profound neurodegeneration resulting from cell death similar to that found in other published Dicer loss studies [22], [47]. However, in another study, the ablation of Dicer in dopaminoceptive neurons resulted not in cell death but led to neuronal dysfunction manifested by a range of phenotypes including ataxia, front and hind limb clasping, reduced brain size, and smaller neurons [48]. The pathways and biological processes in which the miRNAs identified as deregulated in mouse scrapie participate are as yet unknown; only a handful of publications explicitly relate to any of these specific miRNAs. Linking a miRNA to its down-stream gene targets is a major challenge in miRNA biology and only very few genes have been experimentally confirmed as targets for specific miRNAs. However, a variety of bioinformatic processes have been developed which predict potential binding sites within the sequence of gene 3′ UTRs in an effort to identify genes regulated by miRNAs. We used three of the leading algorithms to predict gene targets for our list of disease-specific miRNAs and found that transcriptional regulators, cell signaling molecules and genes involved in cell death and neuronal development are strongly represented amongst the potential targets. If mRNAs are altered in prion disease then miRNAs targeting functionally related genes would be expected to be also de-regulated. We found this to be the case; analysis of these data showed extensive correlation between the gene ontology assignments of de-regulated mRNAs, and putative miRNA targets, in mouse scrapie infected brain, strengthening the hypothesis of miRNA involvement in neurodegenerative disease processes.

Interestingly, some of the largest groups of de-regulated genes in prion disease, for example inflammatory response-related genes were not represented by the putative targets of de-regulated miRNAs. The majority of miRNAs for which functions have been determined appear to exert their effects by down-regulation of specific targets. MiRNAs may be indirect regulators of immune response genes at the level of transcription control. A number of such instances have been described for the regulation of genes involved in transcription by miRNAs as part of a feed back loop. One interesting example is the indirect regulation of the neurotrophic factor BDNF by miR-132 [49]. In this case, miR-132 regulates the translation of transcriptional co-repressor methyl CpG–binding protein 2 (MeCP2), the levels of which are under tight regulation in the brain as both increases and decreases in it expression have been shown to result in neurodevelopmental defects. Additionally mutations in MeCP2 have been linked to the neurodevelopment disorder Rett syndrome underlining its importance. BDNF expression is modulated by MeCP2 as part of a feed-back loop in which miR-132 plays a role, perhaps along with other miRNAs. We identified the up-regulation of miR-328, which also potentially targets MeCP2, perhaps implicating the disruption of neurodevelopmental regulatory circuits is a feature of prion disease.

Given the strong inverse correlation between up-regulated miRNAs and the down regulation of behaviour-related genes, particularly those involved in synaptic function, we investigated the possibility of miRNA mediated regulation of these genes. One gene of interest to us was the transcriptional regulator EGR1. This gene has been shown by us to be consistently down-regulated in scrapie infected mouse brain, detectable at pre-clinical stages of disease [4]. In this study we show that at least *in vitro*, the 3′UTR is a target site of miRNA miR-191, which is up-regulated during scrapie infection. EGR1 has been shown to share a disproportionate number of transcriptional targets with CREB1, also down-regulated in prion disease, suggesting that these two may be functionally related. They have also both been shown to be activated in conjunction with neural activity and indeed disruption of CREB1 expression has been shown to result in hippocampus neurodegeneration [38]. Analysis of the 3′ UTR sequence of CREB1 showed that it shares a number of potential target sites for miRNAs with EGR1. Interestingly, CREB1 has also been shown to be involved in regulation of expression of a number of miRNAs and may be intimately involved in regulatory networks mediating neuronal function and morphogenesis [49]–[53].

In conclusion, we have identified a number of miRNAs that appear to be de-regulated during prion induced neurodegeneration. A number of genes and signaling pathways important in neuronal degeneration in prion diseases are likely regulated at least in part by miRNAs. A co-ordinated de-regulation of miRNAs seen in prion diseases may well be a response to the abnormal accumulation of PrP^Sc^ leading to a pathogenic cascade consisting of compensatory alterations in neurotransmitter receptors, protein degradation pathways and signaling pathways which lead to an ultimate failure of neuronal function. Many miRNAs have been found to show tissue and cell specific expression; it will be interesting to determine the cell types in which the de-regulated miRNAs are expressed as this may shed some light onto those regulatory pathways that lead to cell death in neurons. Given the ability of miRNAs to target multiple genes for expression, and their potential for modulating neuroprotective mechanisms, the prospect of using miRNAs as therapeutics in neurodegenerative diseases is tantalizing. MiRNAs may also have potential for use as biomarkers given our evidence of de-regulation of a prion-specific subset of miRNAs that is distinct from other neurodegenerative diseases and potentially conserved across species.

## Materials and Methods

### Animals

All procedures involving live animals were approved by the Canadian Science Centre for Human and Animal Health Animal Care Committee. All protocols were designed to minimize animal discomfort. Inoculations were performed as previously described [4].

### MicroRNA extraction

MiRNAs were extracted using the *mir*Vana™ miRNA Isolation Kit (Ambion) according to the manufacturer's instructions. This procedure yields an RNA fraction consisting of RNA species of less than 200 bases. Yield and purity of the miRNA enriched fraction was measured by UV absorbance at OD_260/280_.

### MicroRNA microarray procedures

MiRNA microarrays were performed as previously described [23]. Specifically, the Array 900 miRNA RT Kit (Genisphere) was used to prepare labeled cDNA targets for microarray hybridization according to the manufacturer's protocol. Briefly, 250 ng of LMW enriched RNA was used as a template in a 100 µL Poly(A) Tailing and RT reaction containing 1× Reaction Mix, 2.5 mM MnCl_2_, 1 mM ATP, and 4 µL poly A polymerase (*E*-PAP Enzyme). After incubation at 37°C for 15 minutes the reaction was placed on ice and 2 µL of Cy3 or Cy5 reverse transcriptase primer was added. The reaction mix was incubated at 65°C for 10 minutes prior to addition of 23 µL of a second master mix containing per reaction: 10 µL of 5× First Strand Buffer, 5 µL of 0.1 M DTT, 2.5 µL of dNTP Mix, 1 µL of Superase-in RNase Inhibitor, 2 µL of SuperScript II Reverse Transcriptase (200 U) (Invitrogen), and 2.5 µL of nuclease-free water. Subsequently, the reaction was incubated at 42°C for one hour. Finally, 8.75 µL of 0.5 M NaOH/50 mM EDTA and 65°C for 15 minutes was used to inactivate Superscript II. Samples were concentrated to a volume of approximately 10–15 µL, using Microcon YM-10 Centrifugal Filter Devices (Fisher Scientific) according to the manufacturer's protocol.

Tagged-cDNA hybridization followed the protocol outlined in the 900 miRNA RT Kit. A hybridization mixture consisting of the differentially tagged cDNA (10 µL of Cy3-labelled and 10 µL of Cy5-labelled targets) and 2×SDS-based Hybridization Buffer pre-heated to 70°C (20 µL) was mixed and incubated at 75–80°C for 10 minutes, cooled to 50°C until loading and added to the microarray; specifically a 22×40 mm cover slip (mSeries Lifterslip™) (Erie Scientific) was centered over the grids and the preheated hybridization mixture was loaded under the cover slip. Microarrays were incubated overnight (16–20 hours) at 50°C in a dark humidified chamber (Genetix). Following hybridization the cover-slips were removed and the arrays were washed in 2×SSC, 0.2% SDS wash buffer preheated to 42°C for 15 minutes, 2×SSC wash buffer at room temperature for 10–15 minutes, and 0.2×SSC wash buffer at room temperature for 10–15 minutes. Arrays were dried by centrifugation at 1000 rpm for 2–3 minutes and the 3DNA system containing the fluorescent cyanine molecules were hybridized to the arrays; in this case the hybridization mixture contained Cy3 3DNA Capture Reagent (2.5 µL), Cy5 3DNA Capture Reagent (2.5 µL), Nuclease Free Water (15 µL), and 2×SDS-based Hybridization Buffer. The mix was heated to 70°C for 10 minutes, cooled to 62–64°C and hybridized to the arrays for 4 hours at 62–64°C in a dark humidified chamber. Finally, the arrays were washed as previously described [23].

Microarrays were scanned using Agilent G2565AA and Agilent G2565BA Microarray Scanner System (Agilent). Feature extraction was performed using Array-Pro™ analyzer version 4.5 (Media Cybernetics). Data was Log2 transformed and normalized using Lowess subgrid normalization in GeneTraffic Microarray Database and Analysis System (Iobion Informatics). The miRNA microarray related data were submitted to Gene Expression Omnibus (GEO) under accession numbers: [GSE12354].

### Significance Analysis of Microarrays (SAM)

To identify miRNAs that were most significantly different in expression between the scrapie infected mice and age-matched, mock-infected mice, a one-class SAM analysis [54] with a false discovery rate (FDR) of <20% was used. To increase the confidence level of our results, the only miRNAs selected were those with: (1) minimum intensity levels above that of negative control miRNAs spotted on the array, (2) signal-to-background ratio ≥1.5 and (3) readings for ≥50% of the data points was present.

### Quantitative RT-PCR (TaqMan® MiRNA Assays)

Reverse transcriptase reactions were performed using the TaqMan® MicroRNA Reverse Transcription Kit (Applied Biosystems). Each reaction contained 20 ng LMW enriched RNA, 1 mM dNTPs (with dTTP), 1 µL of 3.3 U MultiScribe™ Reverse Transcriptase, 1× Reverse Transcription Buffer, 3.75 U RNase Inhibitor, and 3 µL of RT primer. The reaction was carried out at 16°C for 30 minutes, 42°C for 30 minutes, and 85°C for 5 minutes. Semi-quantitative PCR reactions were performed according to the methodology outlined in the TaqMan® MiRNA Assay Kit (Applied Biosystems). Briefly, each reaction contained TaqMan 1× Universal PCR Master Mix (No AmpErase UNG), 1× TaqMan® MicroRNA Assay Mix, and 1.33 µL of the RT product in a total volume of 20 µL. Each reaction was incubated in an Applied Biosystems 7500 Real Time PCR System in a 96-well plate at 95°C for 10 minutes, followed by 40 cycles of 95°C for 15 sec and 60°C for 1 minute. The threshold cycle (Ct) method was used to determine the relative quantities of each miRNA, and was defined as the fractional cycle number at which the fluorescence passes the fixed threshold. Target miRNA expression was normalized between different samples based on the values determined for let-7a miRNA expression which was found to be routinely consistently expressed in all of the samples tested at the same Ct value (no differential expression between samples). All samples were tested in triplicate throughout. Expression was calculated using the ΔΔCt method: 2^ΔCt Scrapie-infected−ΔCt mock-infected^ with ΔCt = average Ct target miRNA – average Ct let-7a. MiR-16 was used as the positive controls and *C. elegans* and *A. thaliana* miRNAs were used as the negative controls in the experiment.

### MiRNA reporter vector constructs, transfection and dual luciferase reporter assays

To create EGR1 3′UTR luciferase reporter constructs, a ∼1200 bp fragment of the 3′UTR (bp 1885-3072 of NC_000084.5) was cloned downstream of CMV-driven firefly luciferase cassette in pMIR-REPORT vector (Ambion). The 3′UTR of EGR1 was cloned from total mouse cDNA using forward primer 5′ gtacactagtagggaataaaagaaagcaaagggag 3′ and reverse primer 5′ gtacaagcttgaaggatacacaccacata 3′ (Operon Biotechnologies). The FastStart Taq Polymerase PCR kit (Roche) was used to amplify the 3′UTR target sequence. Following purification of PCR products using Kleenspin (Biorad), the amplicon and pMIR-REPORT were digested with SpeI and HindIII restriction enzymes (NEB) at 37°C for 1 hr. Further purification was performed and the products were then ligated at 16°C overnight. Ligation products (2 µL) were then used to chemically transform One Shot TOP10 *E.coli* cells (Invitrogen). Transformed culture (100 µL) was plated on 100 µg/mL LB carbenicillin agar plates and the resulting colonies were screened for the EGR1 insert using the HotStar HiFidelity Polymerase Kit (Qiagen). Selected positive clones were propagated in 100 µg/mL LB agar at 37°C for 24 hrs and the plasmid was extracted using the EndoFree Plasmid MaxiPrep kit (Qiagen). Subcloned inserts were sequenced by the DNA core facility at the National Microbiology Laboratory at the Canadian Science Center for Human and Animal Health and verified by BLASTN search.

The pMIR-REPORT™ miRNA Expression Reporter Vector (Ambion) was used to analyze putative miRNA target binding sites in combination with the Dual-Light Chemiluminescent Reporter Gene Assay System (Applied Biosystems). The siPORT™ NeoFX™ Transfection Agent kit (Ambion) was used to co-transfect HeLa cells with both the reporter vector and mmu-miR-203 or mmu-miR-191 (Ambion). A β-galactosidase reporter control vector was also co-transfected in parallel with the EGR1 vector for normalization of transfection efficiencies. Briefly, 300 ng of the reporter vector and 300 ng of the control vector were diluted in OPTI-MEM media (GIBCO) containing 15, 30, 60 or 100 nM of the synthetic mature miRNA of interest in a final volume of 100 µl. Concurrently, a 6∶100 dilution of the transfection reagent was made in OPTI-MEM and pre-incubated for 10 min at 37°C. Subsequently, 100 µl of the diluted transfection reagent and 100 µL of the reporter plasmid/miRNA dilution were left at room temperature for an additional 10 min. The 200 µL samples were mixed with 2.3 mL of DMEM (GIBCO) containing 2.5×10^5^ Hela cells/mL and then plated in 6-well plates and incubated for an additional 24 hr at 37°C prior to assaying for luciferase activity.

The chemiluminescent reporter gene assay was used for the combined detection of luciferase and β-galactosidase activities according to the manufactures instructions. Luciferase activity was measured using the dual injecting Veritas™ Microplate Luminometer (dual injecting) (Turner Biosystems). Each experimental condition was performed in triplicate or quadruplicate within individual experiments and the results shown represent at least three independent experiments. All results were normalized using simultaneously measured β-galactosidase activity.

### MicroRNA Target prediction

To determine the gene targets of the differentially expressed miRNAs we used three of the leading miRNA target prediction algorithms miRanada (http://microrna.sanger.ac.uk/sequences/) [26], PicTar (http://pictar.mdc-berlin.de/) [27], TargetScan (http://www.targetscan.org/) [28]. To determine genes that were similarly identified by two or more of these algorithms, the online program Matchminer (http://discover.nci.nih.gov/matchminer/index.jsp) was used [55].

### Functional Analysis of MiRNA Target Genes

Functional analysis of MiRNA Target Genes was performed using Ingenuity Pathways Analysis (Ingenuity® Systems). This software analyses lists genes in the context of known biological response and regulatory networks as well as other higher-order response pathways. MiRNA targets that were associated with biological functions in the Ingenuity Pathways Knowledge Base were used in the analysis (focus genes). For all analyses, Fisher's exact test was used to calculate a p-value determining the probability that each biological function assigned to that data set was due to chance alone. Networks of the focus genes were then algorithmically generated based on their connectivity. In the functional network shown in figures 2 and 3, miRNA target genes are represented as nodes, and the biological relationship between two nodes is represented as an edge (line). All edges are supported by at least one published reference or from canonical information. Enrichment of transcription factor motifs in putative miRNA target genes was performed using FatiGO [37]. This tool uses information collected on regulatory motifs from the CisRed [56] and TRANSFAC [57] databases. These motifs were studied in the first 5 Kb of the promoter sequence as described in Al-Sharouf et al. [37].

## Supporting Information

Data S1 ΔΔCT for 114 miRNAs detectable in mouse brain 6 mice infected with scrapie.
(0.25 MB DOC)

Data S2 List of 1282 miRNA target genes that were in consensus between two or more miRNA target prediction programs.
(1.64 MB DOC)
