# Supplementary material for: A miRNA Signature of Prion Induced Neurodegeneration
Source: PLoS One. 2008 Nov 6;3(11):e3652. doi: 10.1371/journal.pone.0003652 (PMC2575400; doi:10.1371/journal.pone.0003652)
Supplement: Data S1 — ΔΔCT for 114 miRNAs detectable in mouse brain 6 mice infected with scrapie. (0.25 MB DOC) [file pone.0003652.s001.doc]

**Data S1.** ∆∆C^T^ for 114 miRNAs detectable in mouse brain 6 mice infected with scrapie.

| MiRNA (Applied Biosystems Probe ID) ∆∆C^T^ | | | | | | | |
| --- | --- | --- | --- | --- | --- | --- | --- |
| abm000002 hsa-miR-9 | 0.226 | 0.5235 | 0.399 | 0.2185 | -1.7295 | -0.0755 |  |
| abm000003 hsa-miR-9* | 0.623 | 0.2235 | 1.45 | -3.9755 | 1.1035 | -4.4935 |  |
| abm000006 hsa-miR-15a | -0.483 | -0.1455 | 3.425 | 0.9255 | 3.425 | 0.5925 |  |
| abm000007 hsa-miR-15b | 0.431 | 0.2315 | -1.234 | -0.6215 | -0.3505 | 0.2545 |  |
| abm000008 hsa-miR-16 | 0.609 | 0.1985 | -0.284 | 0.0195 | 0.0465 | 0.3345 |  |
| abm000008 hsa-miR-16 | 0.419 | 0.3095 | 0.369 | 0.2995 | 0.0095 | 0.1245 |  |
| abm000008 hsa-miR-16 | -0.136 | -0.116 | 0.007 | -2.136 | -0.201 | -1.5245 |  |
| abm000008 hsa-miR-16 | 0.191 | 0.519 | 0.37 | 0.494 | -0.406 | 0.7135 |  |
| abm000010 hsa-miR-17-5p | 0.038 | 0.1145 | -0.536 | -0.6345 | -0.4955 | 0.6505 |  |
| abm000011 hsa-miR-19a | 0.595 | 0.1695 | 0.908 | -0.7165 | -0.6785 | 0.2045 |  |
| abm000013 hsa-miR-20 | 1.226 | -0.1285 | 0.075 | -0.5795 | -0.4325 | 0.6955 |  |
| abm000014 hsa-miR-21 | 1.659 | 0.9425 | 1.496 | 0.1525 | -0.4815 | 0.5815 |  |
| abm000016 hsa-miR-23a | 0.535 | 0.0735 | -0.316 | -0.7135 | 0.0775 | -0.3815 |  |
| abm000017 hsa-miR-23b | 0.115 | -0.0595 | -1.788 | -0.4125 | -0.2105 | -0.4795 |  |
| abm000019 hsa-miR-25 | 0.312 | -0.3555 | -0.394 | -0.9325 | -0.3815 | -0.1355 |  |
| abm000020 hsa-miR-26a | 1.019 | 1.4815 | 0.486 | 0.3055 | 0.4225 | 0.5035 |  |
| abm000021 hsa-miR-26b | 0.664 | 0.9135 | -0.086 | -0.7175 | -0.4015 | -0.3265 |  |
| abm000022 hsa-miR-27a | 1.109 | 0.0865 | 1.454 | -0.2975 | -0.4205 | 0.4635 |  |
| abm000023 hsa-miR-27b | 0.245 | 0.1655 | 0.25 | -0.5895 | -0.2185 | 0.0685 |  |
| abm000025 hsa-miR-29a | 0.018 | 0.9015 | 0.096 | -0.6465 | -0.1685 | -0.5555 |  |
| abm000026 hsa-miR-29b | 0.046 | 2.6175 | 0.768 | -0.2925 | 0.6075 | -0.1275 |  |
| abm000027 hsa-miR-29c | 0.475 | 1.2345 | 0.535 | -0.6225 | 0.4195 | -0.4645 |  |
| abm000028 hsa-miR-30a-3p | 0.702 | 0.2245 | 0.424 | -0.6445 | 0.3095 | -0.9215 |  |
| abm000029 hsa-miR-30b | -0.028 | 0.5245 | -0.491 | -0.4075 | -0.1805 | -0.5215 |  |
| abm000030 hsa-miR-30c | 0.34 | 0.2095 | -0.632 | -0.4155 | -0.2245 | -0.4145 |  |
| abm000031 hsa-miR-30d | 0.56 | -0.1225 | -2.113 | -0.2585 | -0.5155 | -0.1745 |  |
| abm000032 hsa-miR-30e | 0.535 | 0.6875 | 0.44 | -0.7415 | -0.6495 | -0.7455 |  |
| abm000033 hsa-miR-31 | 1.377 | 1.0205 | 0.033 | 0.4855 | 0.3715 | 0.5735 |  |
| abm000036 hsa-miR-34a | 0.746 | -0.3535 | 0.527 | -1.1535 | -0.4005 | -0.7815 |  |
| abm000038 hsa-miR-34c | 0.396 | 0.2415 | 0.852 | 0.0615 | 0.3205 | -0.1635 |  |
| abm000039 hsa-miR-92 | -0.734 | 0.3305 | -2.383 | -0.6705 | -0.1345 | 0.0555 |  |
| abm000042 hsa-miR-96 | 0.204 | -0.7905 | -0.135 | -6.7005 | -0.7225 | -6.6565 |  |
| abm000043 hsa-miR-98 | 1.046 | -0.0575 | -0.017 | -0.9065 | -0.0115 | -0.0845 |  |
| abm000044 hsa-miR-99a | 1.582 | 0.6355 | -0.398 | 0.0735 | -0.3435 | 0.2235 |  |
| abm000046 hsa-miR-100 | 0.547 | -0.0705 | -1.555 | -0.5745 | -0.7385 | -0.6445 |  |
| abm000048 hsa-miR-103 | -0.027 | -0.5105 | -1.656 | -0.6245 | -0.7505 | -0.1805 |  |
| abm000051 hsa-miR-106a | -0.133 | 0.5885 | 1.677 | -0.3325 | -0.6705 | 1.0495 |  |
| abm000053 hsa-miR-107 | -1.981 | 0.0405 | -1.135 | 0.0045 | 0.2425 | -0.2955 |  |
| abm000055 hsa-miR-124a | 0.157 | 0.3475 | 0.184 | -0.0795 | 0.3115 | 0.1045 |  |
| abm000056 hsa-miR-124b | 0.374 | 0.6015 | -0.523 | -0.0995 | 0.3285 | -0.2705 |  |
| abm000057 hsa-miR-125a | 0.339 | -0.6575 | -3.286 | -0.3305 | 0.0595 | -0.3945 |  |
| abm000058 hsa-miR-125b | 0.091 | 0.9285 | -2.74 | -0.0075 | 0.2455 | 0.2015 |  |
| abm000059 hsa-miR-126 | 0.852 | 0.0665 | -0.51 | -1.2635 | -0.5165 | -0.9075 |  |
| abm000060 hsa-miR-127 | 0.587 | 0.0345 | -1.033 | -0.3735 | -0.5045 | -0.0975 |  |
| abm000061 hsa-miR-128a | 0.171 | -0.1265 | 0.493 | -0.5215 | 0.3545 | -0.5355 |  |
| abm000062 hsa-miR-128b | 2.411 | 1.1935 | 1.874 | 0.3695 | 1.4785 | 1.0345 |  |
| abm000063 hsa-miR-129 | 1.3 | 0.7355 | -0.975 | 0.2345 | 0.1905 | 0.2415 |  |
| abm000064 hsa-miR-130a | 0.619 | -0.1355 | -1.505 | -0.3215 | -0.3225 | -0.2125 |  |
| abm000065 hsa-miR-130b | 0.188 | -0.0945 | -0.63 | -0.5965 | -0.8735 | -0.4615 |  |
| abm000066 hsa-miR-132 | 0.535 | -0.1165 | -1.042 | -0.4155 | 0.0235 | -0.7605 |  |
| abm000067 hsa-miR-133a | 0.602 | 0.0825 | -1.545 | -1.2125 | -0.8325 | -1.1675 |  |
| abm000068 hsa-miR-133b | 0.014 | 0.1145 | -1.011 | -0.7155 | 0.3625 | -0.7645 |  |
| abm000069 hsa-miR-134 | -0.057 | 0.1535 | -0.866 | -0.6815 | -0.3145 | -0.4605 |  |
| abm000070 hsa-miR-135a | 0.274 | 0.1715 | 0.637 | -0.5955 | -0.1045 | -0.8135 |  |
| abm000071 hsa-miR-135b | -0.156 | -0.5035 | 0.556 | -1.1045 | -0.4895 | -0.8425 |  |
| abm000073 hsa-miR-137 | 1.069 | 0.1505 | 0.621 | -0.0445 | -0.4705 | -0.2555 |  |
| abm000074 hsa-miR-138 | 0.711 | -0.2215 | 1.506 | 0.0375 | 0.1395 | 0.0575 |  |
| abm000075 hsa-miR-139 | 3.226 | 2.5235 | 1.615 | 2.2685 | 2.3235 | 1.7155 |  |
| abm000076 hsa-miR-140 | 0.156 | -0.1695 | 0.299 | -0.7535 | -0.9685 | -0.4565 |  |
| abm000077 hsa-miR-141 | 0.004 | -0.6765 | 0.679 | -9.1075 | -0.3915 | -7.2955 |  |
| abm000078 hsa-miR-142-3p | 0.669 | -0.4505 | 0.94 | -0.3705 | -0.8925 | 0.0495 |  |
| abm000082 hsa-miR-145 | -0.618 | -0.2535 | -2.008 | -1.1575 | -0.2835 | -1.1265 |  |
| abm000083 hsa-miR-146 | 1.313 | 1.1735 | 0.819 | 1.0715 | 0.2815 | 1.7645 |  |
| abm000087 hsa-miR-149 | 0.363 | 0.2265 | -2.856 | -0.0245 | 0.2505 | 0.1745 |  |
| abm000088 hsa-miR-150 | 0.184 | -0.6335 | -3.15 | -0.3015 | -0.5585 | -0.2305 |  |
| abm000090 hsa-miR-152 | 1.049 | 0.3395 | 2.186 | 1.0765 | 0.3795 | 0.7905 |  |
| abm000092 hsa-miR-154 | 0.357 | 0.207 | 1.24 | -0.494 | 1.163 | -0.0385 |  |
| abm000093 hsa-miR-154* | -0.144 | -0.041 | 1.484 | -0.865 | -0.057 | -0.6035 |  |
| abm000095 hsa-miR-181a | 0.956 | 1.159 | 0.124 | -0.898 | 1.747 | -0.3675 |  |
| abm000096 hsa-miR-181b | 1.097 | 1.492 | -0.956 | 2.065 | 0.438 | 2.2585 |  |
| abm000097 hsa-miR-181c | 0.742 | 1.61 | -0.038 | -2.208 | 0.75 | -1.9105 |  |
| abm000098 hsa-miR-182 | 1.438 | 0.876 | 0.93 | -4.512 | 1.573 | -3.9475 |  |
| abm000102 hsa-miR-185 | 0.063 | 0.333 | 1.143 | -0.735 | 1.498 | -0.1775 |  |
| abm000103 hsa-miR-186 | 0.726 | 0.54 | 0.733 | 1.037 | 1.134 | 1.2325 |  |
| abm000104 hsa-miR-187 | 0.364 | 0.487 | 0.381 | 0.002 | 0.032 | 1.0675 |  |
| abm000108 hsa-miR-191 | 1.533 | 1.903 | 0.704 | 1.659 | 1.283 | 2.7305 |  |
| abm000110 hsa-miR-194 | 0.452 | 0.513 | 0.738 | 1.143 | 0.705 | 0.3785 |  |
| abm000111 hsa-miR-195 | -0.059 | -0.088 | 0.065 | 0.965 | -0.218 | 0.6725 |  |
| abm000115 hsa-miR-199a* | -0.352 | -0.305 | 0.936 | 0.765 | -0.778 | 0.6855 |  |
| abm000117 hsa-miR-199-s | 0.481 | 0.946 | 0.868 | 0.652 | 0.533 | 0.4025 |  |
| abm000118 hsa-miR-200a | 1.364 | 0.858 | 1.558 | -7.289 | 1.407 | -7.3665 |  |
| abm000119 hsa-miR-200b | 2.117 | 1.443 | 1.409 | -10.311 | 1.716 | -9.7155 |  |
| abm000120 hsa-miR-200c | 3.046 | 2.842 | -0.007 | -8.817 | 2.778 | -6.2815 |  |
| abm000121 hsa-miR-203 | 1.393 | 1.726 | 1.833 | 1.119 | 0.575 | 1.3155 |  |
| abm000126 hsa-miR-210 | 1.307 | 0.974 | 0.126 | 0.784 | 0.895 | 1.9165 |  |
| abm000127 hsa-miR-211 | 0.513 | 0.379 | 0.498 | -3.165 | 0.054 | -4.8405 |  |
| abm000128 hsa-miR-213 | 1.05 | 1.281 | 1.683 | 1.713 | 1.267 | 2.1325 |  |
| abm000133 hsa-miR-218 | -0.616 | 0.558 | 0.205 | -0.761 | -1.166 | -0.4615 |  |
| abm000134 hsa-miR-219 | -0.893 | -1.063 | 1.08 | -0.055 | -0.938 | -0.1165 |  |
| abm000136 hsa-miR-221 | 0.577 | 0.638 | 0.687 | 0.925 | 1.188 | 0.9665 |  |
| abm000137 hsa-miR-222 | 0.865 | 0.547 | 0.154 | -1.414 | 1.18 | -0.8185 |  |
| abm000142 hsa-miR-301 | -0.118 | -0.054 | 1.584 | -1.443 | -0.014 | -0.4785 |  |
| abm000149 hsa-miR-320 | 1.582 | 1.528 | -1.833 | 2.921 | 1.838 | 2.8075 |  |
| abm000151 hsa-miR-323 | 0.205 | 0.317 | -0.543 | -1.514 | -0.292 | -1.5375 |  |
| abm000153 hsa-miR-324-5p | 1.052 | 1.582 | 0.561 | -0.059 | 1.126 | 0.1455 |  |
| abm000155 hsa-miR-326 | 1.328 | 1.371 | 0.179 | -4.723 | 0.85 | -7.5925 |  |
| abm000156 hsa-miR-328 | 0.968 | 1.538 | -1.186 | 1.867 | 1.937 | 1.2725 |  |
| abm000158 hsa-miR-331 | 2.079 | 1.822 | -1.071 | 0.758 | 0.92 | 1.0445 |  |
| abm000159 hsa-miR-335 | -0.396 | -0.492 | -0.005 | 0.26 | -0.94 | 0.7435 |  |
| abm000160 hsa-miR-337 | -0.791 | -0.76 | -0.178 | -3.345 | -1.076 | -4.2345 |  |
| abm000161 hsa-miR-338 | -1.355 | -1.582 | -0.661 | -5.084 | ND | -5.3265 |  |
| abm000162 hsa-miR-339 | 1.48 | 1.702 | -0.182 | 2.038 | 1.641 | 2.4495 |  |
| abm000163 hsa-miR-340 | -0.393 | -0.578 | -0.277 | -0.978 | -1.205 | 0.3455 |  |
| abm000164 hsa-miR-342 | 1.917 | 2.092 | 0.766 | 3.294 | 1.762 | 3.6215 |  |
| abm000169 hsa-miR-370 | 0.819 | 1.001 | 0.283 | 1.543 | 0.825 | 0.9735 |  |
| abm000175 hsa-let-7a | -0.191 | 0.0595 | 0.174 | 0.1855 | -0.116 | 0.2435 |  |
| abm000175 hsa-let-7a | 0.191 | -0.0595 | -0.174 | -0.1855 | -0.3325 | -0.2435 |  |
| abm000175 hsa-let-7a | -0.21 | -0.187 | -0.512 | -0.787 | -0.041 | -0.5705 |  |
| abm000175 hsa-let-7a | 0.21 | 0.187 | 0.512 | 0.787 | 0.041 | 0.5705 |  |
| abm000176 hsa-let-7b | 0.897 | 0.989 | 1.617 | 2.648 | 2.016 | 2.6385 |  |
| abm000177 hsa-let-7d | 0.261 | 0.967 | 1.005 | 1.707 | 1.082 | 1.7115 |  |
| abm000178 hsa-let-7e | 0.212 | 0.557 | 0.6 | 1.393 | 1.528 | 0.7045 |  |
| abm000179 hsa-let-7g | -0.276 | 0.997 | 0.376 | 0.415 | -0.059 | 0.0155 |  |
| abm000180 has-let-7i | -0.256 | 0.109 | -0.554 | -0.217 | -0.908 | -0.3115 |  |
