# Supplementary material for: A miRNA Signature of Prion Induced Neurodegeneration
Source: PLoS One. 2008 Nov 6;3(11):e3652. doi: 10.1371/journal.pone.0003652 (PMC2575400; doi:10.1371/journal.pone.0003652)
Supplement: Data S2 — List of 1282 miRNA target genes that were in consensus between two or more miRNA target prediction programs. (1.64 MB DOC) [file pone.0003652.s002.doc]

**Data S2** List of 1282 miRNA target genes that were in consensus between two or more target prediction programs.

| **1110032A04Rik** | mmu-miR-338 | |  |  |  |
| --- | --- | --- | --- | --- | --- |
| **2310004I24Rik** | mmu-miR-370 | |  |  |  |
| **2610101N10Rik** | mmu-miR-139-5p | |  |  |  |
| **2610110G12Rik** | mmu-miR-342-3p | |  |  |  |
| **2610209A20Rik** | mmu-let-7b |  |  |  |  |
| **2700059D21Rik** | mmu-miR-139-5p | |  |  |  |
| **2810030E01Rik** | mmu-let-7b |  |  |  |  |
| **2810410L24Rik** | mmu-miR-203 | |  |  |  |
| **3110048E14Rik** | mmu-let-7b |  |  |  |  |
| **4921505C17Rik** | mmu-miR-342-3p | |  |  |  |
| **4921533L14Rik** | mmu-let-7b |  |  |  |  |
| **4922503N01Rik** | mmu-miR-203 | |  |  |  |
| **5730438N18Rik** | mmu-miR-203 | |  |  |  |
| **5830457O10Rik** | mmu-miR-370 | |  |  |  |
| **6720467C03Rik** | mmu-miR-203 | |  |  |  |
| **9930012K11Rik** | mmu-let-7b | mmu-let-7d |  |  |  |
| **9930028C20Rik** | mmu-let-7b | mmu-let-7d |  |  |  |
| **A2BP1** | mmu-miR-328 | |  |  |  |
| **A730024A03Rik** | mmu-let-7b | mmu-let-7d |  |  |  |
| **AA536749** | mmu-miR-320 | |  |  |  |
| **Aak1** | mmu-let-7b |  |  |  |  |
| **ABCA1** | mmu-miR-128 | |  |  |  |
| **Abcb9** | mmu-let-7b | mmu-let-7d | mmu-miR-128 | |  |
| **Abcc5** | mmu-let-7b | mmu-miR-320 | |  |  |
| **ABCE1** | mmu-miR-203 | |  |  |  |
| **Abhd14a** | mmu-miR-203 | |  |  |  |
| **Abhd2** | mmu-miR-337 | |  |  |  |
| **Abl1** | mmu-miR-203 | |  |  |  |
| **Accn4** | mmu-miR-370 | |  |  |  |
| **Acin1** | mmu-miR-337 | |  |  |  |
| **Aco2** | mmu-miR-203 | |  |  |  |
| **ACTR2** | mmu-miR-338 | |  |  |  |
| **ACVR1** | mmu-miR-338 | |  |  |  |
| **ACVR1** | mmu-miR-128 | |  |  |  |
| **ACVR1B** | mmu-let-7d |  |  |  |  |
| **Adam17** | mmu-miR-338 | |  |  |  |
| **ADAM19** | mmu-miR-128 | |  |  |  |
| **Adamts15** | mmu-let-7b |  |  |  |  |
| **Adamts8** | mmu-let-7b | mmu-let-7d |  |  |  |
| **ADCY1** | mmu-miR-203 | |  |  |  |
| **ADCY2** | mmu-miR-128 | |  |  |  |
| **Adcy3** | mmu-miR-128 | |  |  |  |
| **ADCY6** | mmu-miR-128 | |  |  |  |
| **Adk** | mmu-miR-203 | |  |  |  |
| **Adora2b** | mmu-miR-128 | |  |  |  |
| **Adrb3** | mmu-let-7b | mmu-let-7d |  |  |  |
| **AEBP2** | mmu-miR-139-5p | |  |  |  |
| **Aff1** | mmu-miR-203 | |  |  |  |
| **AFF4** | mmu-miR-203 | mmu-miR-320 | mmu-miR-128 | |  |
| **AGPAT5** | mmu-miR-338 | |  |  |  |
| **AHR** | mmu-miR-203 | |  |  |  |
| **AI118078** | mmu-miR-370 | |  |  |  |
| **AI836003** | mmu-miR-342-3p | |  |  |  |
| **AK2** | mmu-miR-128 | |  |  |  |
| **AKT3** | mmu-miR-337 | mmu-miR-203 | |  |  |
| **AL022779** | mmu-miR-338 | |  |  |  |
| **ALS2CR13** | mmu-miR-139-5p | |  |  |  |
| **Amd1** | mmu-miR-128 | |  |  |  |
| **AMMECR1** | mmu-miR-191 | |  |  |  |
| [**ANK1**](http://www.ncbi.nlm.nih.gov/sites/entrez?Db=gene&Cmd=ShowDetailView&TermToSearch=286) | mmu-miR-128 | |  |  |  |
| **ANK2** | mmu-miR-139-5p | mmu-miR-342-3p | mmu-miR-128 | |  |
| **ANK3** | mmu-miR-370 | |  |  |  |
| **ANKRD13B** | mmu-miR-203 | |  |  |  |
| **ANKRD17** | mmu-miR-342-3p | |  |  |  |
| **ANKRD43** | mmu-miR-128 | |  |  |  |
| **ANP32E** | mmu-miR-203 | |  |  |  |
| **AP1G1** | mmu-miR-337 | mmu-miR-191 | |  |  |
| **AP2B1** | mmu-miR-203 | |  |  |  |
| **AP3M1** | mmu-miR-139-5p | | mmu-miR-320 | |  |
| **Ap4e1** | mmu-miR-338 | |  |  |  |
| **APAF1** | mmu-miR-128 | |  |  |  |
| **Apba1** | mmu-let-7d |  |  |  |  |
| **APBA2** | mmu-miR-128 | |  |  |  |
| **Apbb3** | mmu-let-7b | mmu-let-7d |  |  |  |
| **API5** | mmu-miR-203 | mmu-miR-337 | |  |  |
| **APLP2** | mmu-miR-139-5p | |  |  |  |
| **APPBP2** | mmu-miR-128 | |  |  |  |
| **APRIN** | mmu-miR-128 | |  |  |  |
| **AQP11** | mmu-miR-128 | |  |  |  |
| **Arf1** | mmu-miR-320 | mmu-miR-370 | |  |  |
| **ARF6** | mmu-miR-139-5p | |  |  |  |
| **ARFGEF1** | mmu-miR-128 | |  |  |  |
| **ARHGAP12** | mmu-miR-203 | mmu-miR-128 | |  |  |
| **ARHGAP17** | mmu-miR-337 | |  |  |  |
| **ARHGAP20** | mmu-let-7d |  |  |  |  |
| **ARHGAP21** | mmu-miR-128 | |  |  |  |
| **ARHGAP28** | mmu-let-7d |  |  |  |  |
| **Arhgef10l** | mmu-miR-338 | |  |  |  |
| **ARHGEF11** | mmu-miR-128 | |  |  |  |
| **Arhgef15** | mmu-let-7d |  |  |  |  |
| **ARID2** | mmu-miR-203 | |  |  |  |
| **ARID4B** | mmu-miR-328 | mmu-miR-128 | |  |  |
| **ARID5B** | mmu-miR-320 | |  |  |  |
| **ARL5A** | mmu-miR-338 | |  |  |  |
| **ARL6IP2** | mmu-miR-370 | |  |  |  |
| **ARL8B** | mmu-miR-320 | mmu-miR-128 | |  |  |
| **ARMC8** | mmu-miR-128 | |  |  |  |
| **Armcx2** | mmu-miR-320 | |  |  |  |
| **Arntl** | mmu-miR-203 | |  |  |  |
| **Arpp21** | mmu-let-7d |  |  |  |  |
| [**ARRDC4**](http://www.ncbi.nlm.nih.gov/sites/entrez?Db=gene&Cmd=ShowDetailView&TermToSearch=91947) | mmu-miR-128 | |  |  |  |
| **Arx** | mmu-miR-139-5p | |  |  |  |
| **Ascc2** | mmu-miR-370 | |  |  |  |
| **ASH1L** | mmu-miR-139-5p | |  |  |  |
| **ATAD1** | mmu-miR-139-5p | |  |  |  |
| **ATG9A** | mmu-miR-338 | |  |  |  |
| **ATP11C** | mmu-miR-128 | |  |  |  |
| **Atp2a2** | mmu-let-7d |  |  |  |  |
| **ATP2B1** | mmu-miR-128 | |  |  |  |
| **ATP5G3** | mmu-miR-203 | |  |  |  |
| **Atp6v1a** | mmu-miR-320 | |  |  |  |
| **Atxn1** | mmu-miR-139-5p | |  |  |  |
| **ATXN10** | mmu-miR-128 | |  |  |  |
| **Atxn2l** | mmu-miR-342-3p | |  |  |  |
| **AU040829** | mmu-miR-320 | |  |  |  |
| **AXUD1** | mmu-miR-128 | |  |  |  |
| **AZIN1** | mmu-miR-139-5p | |  |  |  |
| **B230342M21Rik** | mmu-miR-338 | |  |  |  |
| **B4GALT3** | mmu-miR-128 | |  |  |  |
| **Bach2** | mmu-miR-339-5p | |  |  |  |
| **BAG2** | mmu-miR-128 | |  |  |  |
| **Banp** | mmu-miR-320 | |  |  |  |
| **Batf** | mmu-miR-339-5p | |  |  |  |
| **BAZ2B** | mmu-miR-339-5p | mmu-miR-139-5p | mmu-miR-128 | |  |
| **BC023055** | mmu-miR-139-5p | |  |  |  |
| **BC025816** | mmu-miR-320 | |  |  |  |
| **BC038613** | mmu-miR-320 | |  |  |  |
| **BC057552** | mmu-let-7d | mmu-let-7b |  |  |  |
| **Bcdin3** | mmu-miR-338 | mmu-miR-128 | |  |  |
| **Bcdo2** | mmu-miR-338 | |  |  |  |
| **BCL11A** | mmu-miR-128 | |  |  |  |
| **BCL2L1** | mmu-let-7d |  |  |  |  |
| **BCL3** | mmu-miR-128 | |  |  |  |
| **BCL6** | mmu-miR-339-5p | |  |  |  |
| **BCL7A** | mmu-miR-203 | |  |  |  |
| **BCORL1** | mmu-miR-146a | mmu-miR-128 | |  |  |
| **BDNF** | mmu-miR-191 | |  |  |  |
| **BFSP2** | mmu-miR-338 | |  |  |  |
| **BIN3** | mmu-let-7b |  |  |  |  |
| **BIRC6** | mmu-miR-342-3p | |  |  |  |
| **BIVM** | mmu-miR-146a | |  |  |  |
| **BLCAP** | mmu-miR-320 | mmu-miR-339-5p | |  |  |
| **BMF** | mmu-miR-337 | |  |  |  |
| **BMI1** | mmu-miR-139-5p | |  |  |  |
| **BRMS1L** | mmu-miR-191 | |  |  |  |
| **BRSK1** | mmu-miR-128 | |  |  |  |
| **BTBD10** | mmu-miR-337 | |  |  |  |
| **BTBD14B** | mmu-miR-339-5p | |  |  |  |
| **BTBD3** | mmu-miR-203 | |  |  |  |
| **BTG2** | mmu-miR-128 | |  |  |  |
| **Btg3** | mmu-miR-139-5p | |  |  |  |
| **Bzw1** | mmu-let-7b | mmu-let-7d |  |  |  |
| **Bzw2** | mmu-let-7b | mmu-let-7d |  |  |  |
| **C10ORF137** | mmu-miR-128 | |  |  |  |
| **C10ORF56** | mmu-miR-128 | |  |  |  |
| **C11ORF57** | mmu-miR-128 | |  |  |  |
| **C12ORF34** | mmu-miR-128 | |  |  |  |
| **C14ORF24** | mmu-miR-128 | |  |  |  |
| **C15ORF27** | mmu-miR-128 | |  |  |  |
| **C16ORF28** | mmu-miR-128 | |  |  |  |
| **C16ORF5** | mmu-miR-128 | |  |  |  |
| **C17ORF70** | mmu-miR-128 | |  |  |  |
| **C1ORF108** | mmu-miR-128 | |  |  |  |
| **C1ORF144** | mmu-miR-128 | |  |  |  |
| **C1ORF21** | mmu-miR-128 | |  |  |  |
| **C1ORF52** | mmu-miR-128 | |  |  |  |
| **C1ORF9** | mmu-miR-128 | |  |  |  |
| **C20ORF39** | mmu-miR-128 | |  |  |  |
| **C5ORF13** | mmu-miR-128 | |  |  |  |
| **C6ORF60** | mmu-miR-128 | |  |  |  |
| **C9ORF97** | mmu-miR-128 | |  |  |  |
| **CA10** | mmu-miR-128 | |  |  |  |
| **CA12** | mmu-miR-128 | |  |  |  |
| **CA7** | mmu-miR-128 | |  |  |  |
| **CAB39** | mmu-miR-203 | mmu-miR-128 | |  |  |
| **CABLES2** | mmu-miR-128 | |  |  |  |
| **CABP1** | mmu-miR-128 | |  |  |  |
| **CACNA2D2** | mmu-miR-139-5p | |  |  |  |
| **CACNB2** | mmu-miR-339-5p | mmu-miR-128 | |  |  |
| **CACNB4** | mmu-miR-338 | |  |  |  |
| **Cacng1** | mmu-miR-370 | |  |  |  |
| **CALD1** | mmu-miR-320 | |  |  |  |
| **CALN1** | mmu-miR-320 | |  |  |  |
| **CALU** | mmu-let-7b |  |  |  |  |
| **CAMK2G** | mmu-miR-338 | |  |  |  |
| **CAMKK2** | mmu-miR-342-3p | |  |  |  |
| **CAMTA1** | mmu-miR-128 | |  |  |  |
| **CAP1** | mmu-let-7b |  |  |  |  |
| **CAPZA1** | mmu-miR-128 | |  |  |  |
| **CAPZB** | mmu-miR-339-5p | |  |  |  |
| **CASC3** | mmu-miR-128 | |  |  |  |
| **CASK** | mmu-miR-146a | mmu-miR-370 | |  |  |
| **CASKIN1** | mmu-miR-337 | |  |  |  |
| **Casp3** | mmu-let-7b |  |  |  |  |
| **CBFA2T3** | mmu-miR-128 | |  |  |  |
| **CBFB** | mmu-miR-338 | mmu-miR-203 | |  |  |
| **CCDC71** | mmu-miR-128 | |  |  |  |
| **CCM2** | mmu-miR-128 | |  |  |  |
| **CCNC** | mmu-miR-203 | |  |  |  |
| **CCND1** | mmu-let-7b |  |  |  |  |
| **CCNG1** | mmu-miR-203 | mmu-miR-128 | |  |  |
| **CCNJ** | mmu-miR-370 | mmu-miR-128 | |  |  |
| **CCNK** | mmu-miR-370 | mmu-miR-128 | |  |  |
| **CCR7** | mmu-let-7b |  |  |  |  |
| **Cct3** | mmu-miR-128 | |  |  |  |
| **Cct5** | mmu-miR-139-5p | |  |  |  |
| **CD28** | mmu-miR-128 | |  |  |  |
| **CD34** | mmu-miR-128 | |  |  |  |
| **CD4** | mmu-miR-337 | |  |  |  |
| **Cd79b** | mmu-miR-146a | |  |  |  |
| **Cdc2l5** | mmu-miR-320 | |  |  |  |
| **CDC42** | mmu-miR-342-3p | mmu-miR-128 | |  |  |
| **CDC42SE1** | mmu-miR-337 | mmu-miR-370 | |  |  |
| **Cdc91l1** | mmu-miR-328 | |  |  |  |
| **CDH2** | mmu-miR-338 | |  |  |  |
| **Cdh20** | mmu-miR-139-5p | mmu-miR-320 | |  |  |
| **CDH24** | mmu-miR-128 | |  |  |  |
| **CDK6** | mmu-miR-320 | |  |  |  |
| **CDS1** | mmu-miR-128 | |  |  |  |
| **CDV3** | mmu-let-7d |  |  |  |  |
| **Cebpb** | mmu-miR-191 | |  |  |  |
| **Celsr2** | mmu-miR-328 | |  |  |  |
| **Cenpc1** | mmu-miR-203 | |  |  |  |
| **CENTG2** | mmu-miR-203 | |  |  |  |
| **CFL1** | mmu-miR-370 | |  |  |  |
| **CGNL1** | mmu-let-7b |  |  |  |  |
| **Chd4** | mmu-let-7b | mmu-let-7d |  |  |  |
| **Chrd** | mmu-miR-337 | mmu-let-7b | mmu-let-7d |  |  |
| **CHST1** | mmu-miR-128 | |  |  |  |
| **CHST7** | mmu-miR-203 | mmu-miR-328 | |  |  |
| **Cit** | mmu-miR-370 | mmu-miR-128 | |  |  |
| **Cited2** | mmu-miR-203 | mmu-miR-128 | |  |  |
| **Ckb** | mmu-miR-139-5p | |  |  |  |
| **CLASP2** | mmu-miR-203 | |  |  |  |
| **CLCN3** | mmu-miR-128 | |  |  |  |
| **CLDN12** | mmu-let-7d |  |  |  |  |
| **Clmn** | mmu-miR-139-5p | |  |  |  |
| **CLSTN2** | mmu-miR-128 | |  |  |  |
| **CNNM4** | mmu-miR-339-5p | |  |  |  |
| **Cnot6** | mmu-miR-339-5p | mmu-miR-342-3p | mmu-miR-128 | |  |
| **Cnot7** | mmu-miR-320 | mmu-miR-128 | |  |  |
| **CNR1** | mmu-miR-128 | |  |  |  |
| **CNTFR** | mmu-miR-337 | mmu-miR-203 | |  |  |
| **COL11A1** | mmu-miR-139-5p | |  |  |  |
| **Col14a1** | mmu-let-7b |  |  |  |  |
| **COL17A1** | mmu-miR-203 | |  |  |  |
| **COL24A1** | mmu-let-7d |  |  |  |  |
| **Col2a1** | mmu-miR-342-3p | |  |  |  |
| **COL3A1** | mmu-miR-128 | |  |  |  |
| **COL5A2** | mmu-let-7d |  |  |  |  |
| **COPS2** | mmu-miR-320 | |  |  |  |
| **Cops7b** | mmu-miR-203 | |  |  |  |
| **CORO1C** | mmu-miR-128 | |  |  |  |
| **Cpa4** | mmu-let-7b | mmu-let-7d |  |  |  |
| **CPEB1** | mmu-miR-320 | |  |  |  |
| **CPEB3** | mmu-miR-338 | mmu-miR-128 | |  |  |
| **CPEB4** | mmu-miR-128 | |  |  |  |
| **CPLX3** | mmu-miR-342-3p | |  |  |  |
| **Cpne8** |  |  |  |  |  |
| **CPSF4** | mmu-let-7d |  |  |  |  |
| **CPSF6** | mmu-miR-337 | |  |  |  |
| **CREB1** | mmu-miR-337 | mmu-miR-203 | mmu-miR-128 | |  |
| **Creb5** | mmu-miR-320 | |  |  |  |
| **CRSP2** | mmu-miR-128 | |  |  |  |
| **Crtc2** | mmu-miR-328 | |  |  |  |
| **Csda** | mmu-miR-338 | |  |  |  |
| **CSF1** | mmu-miR-128 | |  |  |  |
| **CSN2** | mmu-miR-203 | |  |  |  |
| **CSNK1G1** | mmu-miR-328 | |  |  |  |
| **CTDSP2** | mmu-miR-128 | |  |  |  |
| **CTDSPL** | mmu-miR-128 | |  |  |  |
| **CTDSPL2** | mmu-miR-203 | mmu-miR-128 | |  |  |
| **CTNNB1** | mmu-miR-139-5p | mmu-miR-320 | |  |  |
| **CTNS** | mmu-let-7d |  |  |  |  |
| **Cugbp2** | mmu-miR-339-5p | mmu-miR-342-3p | |  |  |
| **Cul1** | mmu-miR-203 | |  |  |  |
| **Cxcr4** | mmu-miR-139-5p | |  |  |  |
| **CYP39A1** | mmu-miR-128 | |  |  |  |
| **Cyp46a1** | mmu-let-7b | mmu-let-7d |  |  |  |
| **D19Wsu162e** | mmu-miR-342-3p | |  |  |  |
| **D1Ertd161e** | mmu-miR-328 | |  |  |  |
| **DAB2** | mmu-miR-320 | |  |  |  |
| **DACH1** | mmu-miR-338 | mmu-miR-342-3p | |  |  |
| **DAG1** | mmu-miR-320 | |  |  |  |
| **DAZAP1** | mmu-miR-320 | mmu-miR-342-3p | |  |  |
| **DAZAP2** | mmu-miR-128 | |  |  |  |
| **DBN1** | mmu-miR-320 | |  |  |  |
| **DCBLD2** | mmu-miR-337 | mmu-miR-139-5p | |  |  |
| **Dchs1** | mmu-miR-337 | |  |  |  |
| **DCP1A** | mmu-miR-128 | |  |  |  |
| **DCP2** | mmu-miR-128 | |  |  |  |
| **DCUN1D1** | mmu-miR-128 | |  |  |  |
| **DCUN1D3** | mmu-miR-338 | mmu-let-7d | mmu-miR-203 | |  |
| **DCUN1D4** | mmu-miR-128 | |  |  |  |
| **DCX** | mmu-miR-342-3p | mmu-miR-128 | |  |  |
| **DDEF1** | mmu-let-7b |  |  |  |  |
| **Ddit4** | mmu-miR-139-5p | |  |  |  |
| **Ddx19a** | mmu-let-7b |  |  |  |  |
| **DDX3X** | mmu-miR-339-5p | mmu-miR-342-3p | mmu-miR-370 | |  |
| **Ddx3y** | mmu-miR-370 | |  |  |  |
| **Ddx50** | mmu-miR-342-3p | |  |  |  |
| **DDX6** | mmu-miR-203 | mmu-miR-128 | |  |  |
| **Dgcr2** | mmu-miR-203 | mmu-miR-342-3p | |  |  |
| **DGKZ** | mmu-miR-203 | |  |  |  |
| **DHX15** | mmu-miR-320 | mmu-miR-139-5p | |  |  |
| **Dhx57** | mmu-let-7b |  |  |  |  |
| **DIRAS2** | mmu-miR-128 | |  |  |  |
| **DKK2** | mmu-miR-128 | |  |  |  |
| **Dlg5** | mmu-miR-203 | |  |  |  |
| **DLGAP1** | mmu-miR-146a | mmu-let-7b |  |  |  |
| **Dlgap4** | mmu-miR-320 | |  |  |  |
| **DLL4** | mmu-miR-128 | |  |  |  |
| **DMD** | mmu-miR-139-5p | |  |  |  |
| **DMTF1** | mmu-miR-337 | |  |  |  |
| **DNAJC13** | mmu-miR-128 | |  |  |  |
| **Dnajc5** | mmu-miR-139-5p | |  |  |  |
| **DNAJC6** | mmu-miR-342-3p | |  |  |  |
| **Dnajc7** | mmu-miR-339-5p | |  |  |  |
| **Dnd1** | mmu-miR-370 | |  |  |  |
| **DNER** | mmu-miR-320 | |  |  |  |
| **Dnm1** | mmu-miR-337 | |  |  |  |
| **DNM3** | mmu-miR-337 | |  |  |  |
| **DNMT3B** | mmu-miR-370 | |  |  |  |
| **Dot1l** | mmu-let-7b | mmu-let-7d | mmu-miR-128 | |  |
| **Dpp3** | mmu-let-7b | mmu-let-7d |  |  |  |
| **Dr1** | mmu-miR-203 | |  |  |  |
| **DSCR1L1** | mmu-miR-128 | |  |  |  |
| **DTNA** | mmu-miR-320 | mmu-miR-128 | |  |  |
| **DTX1** | mmu-miR-128 | |  |  |  |
| **Dtx2** | mmu-let-7b | mmu-let-7d |  |  |  |
| **Duox1** | mmu-miR-338 | |  |  |  |
| **DUSP5** | mmu-miR-128 | |  |  |  |
| **Dusp6** | mmu-let-7d |  |  |  |  |
| **DVL2** | mmu-miR-128 | |  |  |  |
| **DYRK2** | mmu-miR-338 | |  |  |  |
| **E230022H04Rik** | mmu-miR-191 | |  |  |  |
| **E2f1** | mmu-miR-320 | |  |  |  |
| **E2F3** | mmu-miR-128 | |  |  |  |
| **E2f5** | mmu-let-7b | mmu-let-7d |  |  |  |
| **E2F6** | mmu-let-7d | mmu-let-7b |  |  |  |
| **EBF3** | mmu-miR-337 | mmu-miR-128 | |  |  |
| **ECE2** | mmu-miR-128 | |  |  |  |
| **EDAR** | mmu-miR-128 | |  |  |  |
| **EDD1** | mmu-miR-128 | |  |  |  |
| **EDN1** | mmu-miR-203 | |  |  |  |
| **EDNRA** | mmu-miR-128 | |  |  |  |
| **EEA1** | mmu-miR-342-3p | |  |  |  |
| **Eed** | mmu-miR-337 | |  |  |  |
| **EFCBP1** | mmu-miR-337 | mmu-miR-203 | |  |  |
| **EFHA2** | mmu-miR-128 | |  |  |  |
| **EGFR** | mmu-miR-128 | |  |  |  |
| **EGLN1** | mmu-miR-203 | |  |  |  |
| **EGR1** | mmu-miR-191 | mmu-miR-203 | |  |  |
| **EHD3** | mmu-miR-128 | |  |  |  |
| **EIF2C2** | mmu-miR-128 | |  |  |  |
| **EIF2S1** | mmu-miR-337 | |  |  |  |
| **EIF3S1** | mmu-miR-320 | |  |  |  |
| **EIF4B** | mmu-miR-139-5p | |  |  |  |
| **EIF4E** | mmu-miR-337 | |  |  |  |
| **EIF4G2** | mmu-miR-146a | mmu-miR-139-5p | mmu-let-7b | mmu-let-7d |  |
| **ELAVL1** | mmu-miR-146a | |  |  |  |
| **ELAVL2** | mmu-miR-337 | mmu-miR-139-5p | mmu-miR-203 | mmu-miR-342-3p | |
| **ELL2** | mmu-miR-128 | |  |  |  |
| **ELMO1** | mmu-miR-128 | |  |  |  |
| **ELOVL4** | mmu-miR-128 | |  |  |  |
| **ELOVL5** | mmu-miR-203 | |  |  |  |
| **ELOVL6** | mmu-miR-128 | |  |  |  |
| **EMILIN2** | mmu-miR-339-5p | |  |  |  |
| **EN1** | mmu-miR-338 | |  |  |  |
| **EN2** | mmu-miR-203 | mmu-miR-128 | |  |  |
| **EP300** | mmu-miR-203 | mmu-miR-342-3p | |  |  |
| **EPB41L1** | mmu-miR-128 | |  |  |  |
| **EPC2** | mmu-miR-203 | |  |  |  |
| **EPHA4** | mmu-miR-339-5p | |  |  |  |
| **EPHB2** | mmu-miR-203 | mmu-miR-128 | |  |  |
| **EPHB3** | mmu-miR-339-5p | mmu-miR-328 | |  |  |
| **ESR1** | mmu-miR-203 | |  |  |  |
| **ESRRA** | mmu-miR-328 | |  |  |  |
| **ESRRG** | mmu-miR-139-5p | mmu-miR-320 | |  |  |
| **ETS1** | mmu-miR-338 | mmu-miR-139-5p | |  |  |
| **ETV1** | mmu-miR-203 | |  |  |  |
| **ETV6** | mmu-miR-328 | |  |  |  |
| **EVI5** | mmu-miR-128 | |  |  |  |
| **EYA1** | mmu-miR-128 | |  |  |  |
| **EYA4** | mmu-miR-320 | mmu-miR-128 | |  |  |
| **Ezh2** | mmu-let-7b | mmu-let-7d |  |  |  |
| **F11R** | mmu-miR-338 | |  |  |  |
| **F13A1** | mmu-miR-128 | |  |  |  |
| **FADS1** | mmu-miR-128 | |  |  |  |
| **FAM105B** | mmu-miR-128 | |  |  |  |
| **FAM78A** | mmu-miR-128 | |  |  |  |
| **FAM84B** | mmu-miR-128 | |  |  |  |
| **Fbf1** | mmu-miR-339-5p | |  |  |  |
| **FBLN2** | mmu-miR-128 | |  |  |  |
| **Fbn2** | mmu-miR-139-5p | |  |  |  |
| **Fbxl10** | mmu-miR-146a | |  |  |  |
| **Fbxl12** | mmu-let-7d |  |  |  |  |
| **FBXL3** | mmu-miR-342-3p | |  |  |  |
| **Fbxl5** | mmu-miR-203 | |  |  |  |
| **FBXL7** | mmu-miR-139-5p | |  |  |  |
| **Fbxo32** | mmu-let-7b |  |  |  |  |
| **FBXO33** | mmu-miR-337 | mmu-miR-338 | mmu-miR-342-3p | mmu-miR-128 | |
| **Fbxo43** | mmu-miR-203 | |  |  |  |
| **Fbxo46** | mmu-miR-370 | |  |  |  |
| **Fbxw7** | mmu-miR-338 | mmu-miR-128 | |  |  |
| **FCHO2** | mmu-miR-139-5p | |  |  |  |
| **Fgf7** | mmu-miR-370 | |  |  |  |
| **Fkbp10** | mmu-miR-328 | |  |  |  |
| **Fkbp1a** | mmu-miR-338 | mmu-miR-320 | |  |  |
| **FKBP5** | mmu-miR-203 | |  |  |  |
| **FLJ31818** | mmu-miR-128 | |  |  |  |
| **Flna** | mmu-miR-146a | |  |  |  |
| **Flrt3** | mmu-miR-128 | |  |  |  |
| **FLT1** | mmu-miR-339-5p | |  |  |  |
| **Fntb** | mmu-let-7d |  |  |  |  |
| **Fos** | mmu-miR-338 | mmu-miR-139-5p | |  |  |
| **FOSB** | mmu-miR-338 | mmu-miR-203 | mmu-miR-370 | mmu-miR-128 | |
| **Foxk2** | mmu-miR-203 | |  |  |  |
| **FOXO1A** | mmu-miR-139-5p | mmu-miR-128 | |  |  |
| **Foxp1** | mmu-miR-337 | |  |  |  |
| **FOXP2** | mmu-miR-128 | |  |  |  |
| **FOXP4** | mmu-miR-128 | |  |  |  |
| **FRMD4A** | mmu-miR-139-5p | mmu-miR-128 | |  |  |
| **FRS3** | mmu-miR-128 | |  |  |  |
| **FUBP3** | mmu-miR-139-5p | |  |  |  |
| **Fuk** | mmu-miR-146a | |  |  |  |
| **FURIN** | mmu-miR-128 | |  |  |  |
| **Fut8** | mmu-miR-342-3p | |  |  |  |
| **FXR2** | mmu-miR-128 | |  |  |  |
| **Fzd5** | mmu-miR-191 | |  |  |  |
| **FZD7** | mmu-miR-128 | |  |  |  |
| **GABARAPL1** | mmu-miR-203 | |  |  |  |
| **Gabpb1** | mmu-miR-338 | |  |  |  |
| **GABRA1** | mmu-miR-320 | |  |  |  |
| **GABRB2** | mmu-miR-203 | |  |  |  |
| **GABRB3** | mmu-miR-203 | |  |  |  |
| **GAD1** | mmu-miR-128 | |  |  |  |
| **GAD2** | mmu-miR-128 | |  |  |  |
| **Galnt3** | mmu-miR-139-5p | |  |  |  |
| **GALNT3** | mmu-miR-128 | |  |  |  |
| **GALNT7** | mmu-miR-128 | |  |  |  |
| **GAP43** | mmu-miR-191 | |  |  |  |
| **GAS2** | mmu-miR-139-5p | |  |  |  |
| **GATA2** | mmu-miR-128 | |  |  |  |
| **GATA6** | mmu-miR-338 | |  |  |  |
| **Gatad2a** | mmu-miR-128 | |  |  |  |
| **Gatm** | mmu-let-7b | mmu-let-7d |  |  |  |
| **Gcc1** | mmu-miR-128 | |  |  |  |
| **GDF10** | mmu-miR-139-5p | |  |  |  |
| **GFPT2** | mmu-miR-128 | |  |  |  |
| **Gga3** | mmu-let-7b | mmu-let-7d |  |  |  |
| **Gipc1** | mmu-let-7b |  |  |  |  |
| **GJA1** | mmu-miR-342-3p | |  |  |  |
| **Gltp** | mmu-miR-128 | |  |  |  |
| **Gmps** | mmu-miR-337 | |  |  |  |
| **GNAI2** | mmu-miR-339-5p | |  |  |  |
| **GNAO1** | mmu-miR-139-5p | |  |  |  |
| **GNB2** | mmu-miR-128 | |  |  |  |
| **GNG12** | mmu-miR-128 | |  |  |  |
| **Gng5** | mmu-let-7b | mmu-let-7d |  |  |  |
| **GOLPH2** | mmu-miR-128 | |  |  |  |
| **Golt1b** | mmu-miR-320 | |  |  |  |
| **GOSR1** | mmu-miR-337 | |  |  |  |
| **Gosr2** | mmu-miR-342-3p | |  |  |  |
| **GPAM** | mmu-miR-128 | |  |  |  |
| **Gpatc1** | mmu-miR-203 | |  |  |  |
| **Gpatc2** | mmu-let-7b |  |  |  |  |
| **GPBP1** | mmu-miR-320 | |  |  |  |
| **GPBP1L1** | mmu-miR-342-3p | |  |  |  |
| **GPM6A** | mmu-miR-328 | |  |  |  |
| **Gpr156** | mmu-let-7b |  |  |  |  |
| **Gpr45** | mmu-let-7b |  |  |  |  |
| **Gpr62** | mmu-miR-370 | |  |  |  |
| **GPR64** | mmu-miR-342-3p | |  |  |  |
| **Gpr85** | mmu-miR-203 | |  |  |  |
| **GRAMD1C** | mmu-miR-203 | |  |  |  |
| **GRB2** | mmu-miR-128 | |  |  |  |
| **GREM1** | mmu-miR-128 | |  |  |  |
| **GRHL3** | mmu-miR-203 | |  |  |  |
| **GRIA3** | mmu-miR-146a | |  |  |  |
| **Gria4** | mmu-miR-339-5p | |  |  |  |
| **Grid2ip** | mmu-let-7b |  |  |  |  |
| **Grm3** | mmu-miR-337 | mmu-miR-339-5p | |  |  |
| **Gspt1** | mmu-miR-320 | mmu-miR-128 | |  |  |
| **GSPT2** | mmu-miR-320 | |  |  |  |
| **Gtf2a1** | mmu-miR-342-3p | |  |  |  |
| **Gtf2a2** | mmu-miR-128 | |  |  |  |
| **GTF2H1** | mmu-miR-337 | |  |  |  |
| **H2afv** | mmu-miR-139-5p | |  |  |  |
| **H2AFX** | mmu-miR-337 | |  |  |  |
| **H3f3a** | mmu-miR-203 | |  |  |  |
| **H3F3B** | mmu-miR-128 | |  |  |  |
| **Hand1** | mmu-let-7b |  |  |  |  |
| **Hand2** | mmu-miR-203 | |  |  |  |
| **HAO1** | mmu-miR-128 | |  |  |  |
| **HAP1** | mmu-miR-339-5p | mmu-miR-328 | |  |  |
| **HAPLN1** | mmu-miR-128 | |  |  |  |
| **HAS3** | mmu-miR-128 | |  |  |  |
| **HBEGF** | mmu-miR-128 | |  |  |  |
| **HCN4** | mmu-miR-128 | |  |  |  |
| **Hdac2** | mmu-miR-337 | |  |  |  |
| **HDGF** | mmu-miR-139-5p | |  |  |  |
| **HDLBP** | mmu-let-7d |  |  |  |  |
| **HECTD1** | mmu-miR-128 | |  |  |  |
| **HECTD2** | mmu-miR-320 | |  |  |  |
| **Helz** | mmu-miR-320 | |  |  |  |
| **HIATL1** | mmu-miR-338 | |  |  |  |
| **HIC2** | mmu-miR-146a | |  |  |  |
| **HIVEP2** | mmu-miR-320 | |  |  |  |
| **Hlx1** | mmu-miR-128 | |  |  |  |
| **HMGA2** | mmu-let-7d |  |  |  |  |
| **HMGB3** | mmu-miR-128 | |  |  |  |
| **Hmox2** | mmu-miR-338 | |  |  |  |
| **HNRPA1** | mmu-miR-339-5p | |  |  |  |
| **Hnrpd** | mmu-miR-146a | |  |  |  |
| **HNRPF** | mmu-miR-139-5p | |  |  |  |
| **HNRPF** | mmu-miR-128 | |  |  |  |
| **Hnrpk** | mmu-miR-339-5p | |  |  |  |
| **Hnrpl** | mmu-miR-203 | mmu-miR-370 | |  |  |
| **HOMER3** | mmu-miR-203 | |  |  |  |
| **HOXA10** | mmu-miR-128 | |  |  |  |
| **HOXA11** | mmu-miR-339-5p | |  |  |  |
| **HOXA13** | mmu-miR-128 | |  |  |  |
| **HOXA3** | mmu-miR-338 | |  |  |  |
| **Hoxb8** | mmu-miR-328 | mmu-miR-128 | |  |  |
| **HOXC6** | mmu-miR-128 | |  |  |  |
| **Hoxd1** | mmu-let-7d |  |  |  |  |
| **HRB** | mmu-miR-342-3p | |  |  |  |
| **Hspb6** | mmu-miR-320 | |  |  |  |
| **Hspb8** | mmu-miR-337 | |  |  |  |
| **HTR4** | mmu-let-7d |  |  |  |  |
| **ID2** | mmu-miR-128 | |  |  |  |
| **Id4** | mmu-miR-342-3p | |  |  |  |
| **IGF1R** | mmu-miR-320 | |  |  |  |
| **Igf2bp1** | mmu-miR-370 | |  |  |  |
| **IGF2BP3** | mmu-miR-320 | mmu-miR-342-3p | |  |  |
| **Ihpk2** | mmu-miR-337 | |  |  |  |
| **Ikbke** | mmu-let-7d | mmu-let-7b |  |  |  |
| **Il10** | mmu-let-7d | mmu-let-7b |  |  |  |
| **IL17RD** | mmu-miR-337 | |  |  |  |
| **Il6** | mmu-let-7b |  |  |  |  |
| **INA** | mmu-miR-203 | mmu-miR-320 | |  |  |
| **Inadl** | mmu-miR-370 | |  |  |  |
| **ING5** | mmu-miR-128 | |  |  |  |
| **INOC1** | mmu-miR-342-3p | mmu-miR-370 | |  |  |
| **INSIG1** | mmu-miR-203 | |  |  |  |
| **INSM1** | mmu-miR-338 | mmu-miR-128 | |  |  |
| **INSR** | mmu-miR-128 | |  |  |  |
| **IPO7** | mmu-miR-128 | |  |  |  |
| **Irak1** | mmu-miR-146a | |  |  |  |
| **IRF2** | mmu-miR-338 | |  |  |  |
| **IRF4** | mmu-miR-128 | |  |  |  |
| **IRS1** | mmu-miR-337 | mmu-miR-128 | |  |  |
| **ISL1** | mmu-miR-203 | mmu-miR-128 | |  |  |
| **ITGA5** | mmu-miR-328 | mmu-miR-128 | |  |  |
| **ITPKC** | mmu-miR-128 | |  |  |  |
| **ITSN2** | mmu-miR-128 | |  |  |  |
| **IXL** | mmu-miR-370 | |  |  |  |
| **Jmjd1a** | mmu-let-7b |  |  |  |  |
| **JMJD1A** | mmu-miR-128 | |  |  |  |
| **Jmjd3** | mmu-miR-342-3p | mmu-miR-139-5p | |  |  |
| **Josd2** | mmu-let-7b |  |  |  |  |
| **Jph1** | mmu-miR-337 | |  |  |  |
| **Jundm2** | mmu-miR-139-5p | |  |  |  |
| **KBTBD2** | mmu-miR-339-5p | mmu-miR-139-5p | |  |  |
| **KBTBD8** | mmu-miR-139-5p | mmu-miR-128 | |  |  |
| **KCNA6** | mmu-miR-128 | |  |  |  |
| **KCNAB1** | mmu-miR-128 | |  |  |  |
| **Kcnab2** | mmu-miR-339-5p | |  |  |  |
| **KCND2** | mmu-miR-338 | |  |  |  |
| **Kcnh3** | mmu-miR-339-5p | |  |  |  |
| **KCNJ11** | mmu-miR-338 | |  |  |  |
| **KCNK10** | mmu-miR-128 | |  |  |  |
| **Kctd15** | mmu-miR-146a | |  |  |  |
| **KCTD5** | mmu-miR-342-3p | |  |  |  |
| **Khdrbs2** | mmu-miR-337 | |  |  |  |
| **KIAA1012** | mmu-miR-128 | |  |  |  |
| **KIAA1033** | mmu-miR-128 | |  |  |  |
| **KIAA1199** | mmu-miR-128 | |  |  |  |
| **KIAA1212** | mmu-miR-128 | |  |  |  |
| **KIAA1737** | mmu-miR-128 | |  |  |  |
| **KIAA1787** | mmu-miR-128 | |  |  |  |
| **KIF21B** | mmu-let-7d | mmu-miR-203 | |  |  |
| **KIF2A** | mmu-miR-338 | mmu-miR-203 | |  |  |
| **KIFC2** | mmu-miR-370 | |  |  |  |
| **Klc2** | mmu-miR-370 | |  |  |  |
| **KLF4** | mmu-miR-128 | |  |  |  |
| **Klf8** | mmu-let-7b | mmu-let-7d |  |  |  |
| **Klhl6** | mmu-let-7b | mmu-let-7d |  |  |  |
| **KPNA3** | mmu-miR-128 | |  |  |  |
| **KPNA4** | mmu-miR-139-5p | |  |  |  |
| **KPNB1** | mmu-miR-342-3p | mmu-miR-203 | |  |  |
| **Krt2-1** | mmu-miR-203 | |  |  |  |
| **KTN1** | mmu-miR-139-5p | |  |  |  |
| **KUA-UEV** | mmu-miR-128 | |  |  |  |
| **LBH** | mmu-miR-128 | |  |  |  |
| **LDHB** | mmu-miR-139-5p | |  |  |  |
| **LETMD1** | mmu-miR-128 | |  |  |  |
| **LHFPL3** | mmu-miR-128 | |  |  |  |
| **LIMD1** | mmu-let-7b |  |  |  |  |
| **LIMK1** | mmu-miR-128 | |  |  |  |
| **Lin28** | mmu-miR-370 | mmu-miR-146a | mmu-miR-128 | |  |
| **LITAF** | mmu-miR-128 | |  |  |  |
| **LMBR1L** | mmu-miR-128 | |  |  |  |
| **LMTK2** | mmu-miR-128 | |  |  |  |
| **LOC153222** | mmu-miR-128 | |  |  |  |
| **LOC339745** | mmu-miR-128 | |  |  |  |
| **LOC399947** | mmu-miR-128 | |  |  |  |
| **LONRF1** | mmu-miR-128 | |  |  |  |
| **LOXL1** | mmu-miR-339-5p | |  |  |  |
| **Loxl3** | mmu-let-7d |  |  |  |  |
| **LPP** | mmu-miR-203 | |  |  |  |
| **Lrig3** | mmu-let-7d | mmu-let-7b |  |  |  |
| **LRRC15** | mmu-miR-146a | |  |  |  |
| **LRRC59** | mmu-miR-338 | |  |  |  |
| **Lrrn3** | mmu-miR-203 | mmu-miR-139-5p | |  |  |
| **LSM1** | mmu-miR-128 | |  |  |  |
| **LSM12** | mmu-miR-128 | |  |  |  |
| **LTBP1** | mmu-miR-203 | mmu-miR-128 | |  |  |
| **LUC7L2** | mmu-miR-320 | |  |  |  |
| **MAF** | mmu-miR-338 | mmu-miR-139-5p | |  |  |
| **Mafb** | mmu-miR-338 | |  |  |  |
| **MAFK** | mmu-miR-203 | |  |  |  |
| **Magi1** | mmu-miR-320 | |  |  |  |
| **MAN2A1** | mmu-miR-128 | |  |  |  |
| **MANEAL** | mmu-miR-128 | |  |  |  |
| **Mansc1** | mmu-miR-370 | |  |  |  |
| **Map2k3** | mmu-miR-370 | |  |  |  |
| **MAP2K4** | mmu-miR-128 | |  |  |  |
| **MAP2K7** | mmu-miR-370 | mmu-miR-128 | |  |  |
| **Map3k1** | mmu-let-7b |  |  |  |  |
| **MAP3K8** | mmu-miR-146a | mmu-miR-370 | |  |  |
| **Map4k3** | mmu-miR-338 | mmu-miR-203 | |  |  |
| **MAP4K5** | mmu-miR-128 | |  |  |  |
| **Mapk1** | mmu-miR-320 | |  |  |  |
| **MAPK14** | mmu-miR-128 | |  |  |  |
| **Mapk8ip3** | mmu-miR-320 | |  |  |  |
| **MAPK9** | mmu-miR-203 | |  |  |  |
| **MARCKS** | mmu-miR-128 | |  |  |  |
| **Mark2** | mmu-miR-328 | |  |  |  |
| **MAT2A** | mmu-miR-139-5p | mmu-miR-146a | mmu-miR-320 | |  |
| **MATN3** | mmu-miR-128 | |  |  |  |
| **MATR3** | mmu-miR-203 | |  |  |  |
| **MAX** | mmu-miR-328 | |  |  |  |
| **MBD6** | mmu-miR-337 | mmu-miR-203 | |  |  |
| **MBNL1** | mmu-miR-203 | mmu-miR-328 | |  |  |
| **MBNL2** | mmu-miR-203 | mmu-miR-128 | |  |  |
| **MECP2** | mmu-miR-328 | |  |  |  |
| **MEGF11** | mmu-miR-128 | |  |  |  |
| **MEIS2** | mmu-miR-128 | |  |  |  |
| **MESDC1** | mmu-miR-128 | |  |  |  |
| **MFSD2** | mmu-miR-128 | |  |  |  |
| **MGAT1** | mmu-miR-128 | |  |  |  |
| **MGC42367** | mmu-miR-128 | |  |  |  |
| **MIER1** | mmu-miR-320 | |  |  |  |
| [**MIER2**](http://www.ncbi.nlm.nih.gov/sites/entrez?Db=gene&Cmd=ShowDetailView&TermToSearch=54531) | mmu-miR-128 | |  |  |  |
| **MIPOL1** | mmu-miR-128 | |  |  |  |
| **Mllt10** | mmu-let-7b |  |  |  |  |
| **MLLT3** | mmu-miR-320 | |  |  |  |
| **MLLT7** | mmu-miR-128 | |  |  |  |
| **MLSTD2** | mmu-miR-128 | |  |  |  |
| **MMD** | mmu-miR-128 | |  |  |  |
| **MME** | mmu-miR-128 | |  |  |  |
| **MNT** | mmu-let-7d | mmu-miR-328 | mmu-miR-128 | |  |
| **Morf4l1** | mmu-miR-338 | mmu-miR-191 | |  |  |
| **Morf4l2** | mmu-miR-338 | mmu-miR-203 | |  |  |
| **MOSPD3** | mmu-miR-128 | |  |  |  |
| **MPP2** | mmu-miR-128 | |  |  |  |
| **MPZ** | mmu-miR-203 | |  |  |  |
| **Mrfap1** | mmu-miR-342-3p | |  |  |  |
| **Mrg2** | mmu-let-7b | mmu-let-7d |  |  |  |
| **MSI1** | mmu-miR-128 | |  |  |  |
| **MTDH** | mmu-miR-128 | |  |  |  |
| **MTMR10** | mmu-miR-128 | |  |  |  |
| **Mxi1** | mmu-miR-320 | |  |  |  |
| **Mybl1** | mmu-miR-139-5p | |  |  |  |
| **Myef2** | mmu-miR-203 | |  |  |  |
| **Mylk2** | mmu-miR-342-3p | |  |  |  |
| **Myo10** | mmu-miR-320 | |  |  |  |
| **MYO1C** | mmu-miR-339-5p | |  |  |  |
| **MYO1F** | mmu-let-7d |  |  |  |  |
| **Myrip** | mmu-let-7d |  |  |  |  |
| **MYT1** | mmu-miR-146a | mmu-miR-128 | |  |  |
| **NAB1** | mmu-miR-128 | |  |  |  |
| **NAP1L1** | mmu-let-7b |  |  |  |  |
| **NAP1L5** | mmu-miR-320 | |  |  |  |
| **NARG1** | mmu-miR-320 | mmu-miR-342-3p | mmu-miR-370 | |  |
| **Narg1** | mmu-miR-128 | |  |  |  |
| **NARG1** | mmu-miR-128 | |  |  |  |
| **NAV2** | mmu-miR-128 | |  |  |  |
| **NAV3** | mmu-miR-128 | |  |  |  |
| **Nbea** | mmu-miR-342-3p | |  |  |  |
| **NCALD** | mmu-miR-370 | |  |  |  |
| **Ncam1** | mmu-miR-128 | |  |  |  |
| **NCDN** | mmu-miR-320 | mmu-miR-370 | |  |  |
| **NCK2** | mmu-miR-339-5p | |  |  |  |
| **Ncoa1** | mmu-miR-128 | |  |  |  |
| **Ncoa4** | mmu-miR-337 | |  |  |  |
| **NCOA7** | mmu-miR-128 | |  |  |  |
| **NDRG4** | mmu-miR-139-5p | |  |  |  |
| **Ndst1** | mmu-miR-191 | mmu-miR-128 | |  |  |
| **Ndst2** | mmu-let-7d | mmu-let-7b |  |  |  |
| **NDUFS4** | mmu-miR-128 | |  |  |  |
| **NEDD4** | mmu-miR-128 | |  |  |  |
| **NEGR1** | mmu-miR-320 | |  |  |  |
| **Nek2** | mmu-miR-128 | |  |  |  |
| **NEK6** | mmu-miR-128 | |  |  |  |
| **Neo1** | mmu-miR-128 | |  |  |  |
| **NEUROD6** | mmu-miR-128 | |  |  |  |
| **Nfatc3** | mmu-miR-370 | |  |  |  |
| **NFE2L2** | mmu-miR-128 | |  |  |  |
| **NGFR** | mmu-miR-128 | |  |  |  |
| **Ngfrap1** | mmu-miR-128 | |  |  |  |
| **NHLH2** | mmu-miR-342-3p | mmu-miR-128 | |  |  |
| **NHS** | mmu-miR-128 | |  |  |  |
| **Nlgn2** | mmu-miR-370 | |  |  |  |
| **Nme6** | mmu-let-7b | mmu-let-7d |  |  |  |
| **NME7** | mmu-miR-139-5p | |  |  |  |
| **NMNAT2** | mmu-miR-203 | |  |  |  |
| **Nnat** | mmu-miR-339-5p | |  |  |  |
| **NONO** | mmu-miR-320 | |  |  |  |
| **NOVA1** | mmu-miR-128 | |  |  |  |
| **NPAS2** | mmu-miR-320 | |  |  |  |
| **Npas4** | mmu-miR-146a | |  |  |  |
| **Npepl1** | mmu-let-7b |  |  |  |  |
| **NPEPPS** | mmu-miR-342-3p | mmu-miR-128 | |  |  |
| **Nppc** | mmu-miR-203 | |  |  |  |
| **NPTX1** | mmu-miR-128 | |  |  |  |
| **NPTX2** | mmu-miR-128 | |  |  |  |
| **NR2F6** | mmu-miR-128 | |  |  |  |
| **NR3C1** | mmu-miR-328 | |  |  |  |
| **NR5A2** | mmu-miR-128 | |  |  |  |
| **NRBF2** | mmu-miR-128 | |  |  |  |
| **NRIP1** | mmu-miR-128 | |  |  |  |
| **Nrk** | mmu-miR-139-5p | mmu-miR-128 | |  |  |
| **NRL** | mmu-miR-338 | |  |  |  |
| **Nrp1** | mmu-miR-338 | |  |  |  |
| **NRP2** | mmu-miR-128 | |  |  |  |
| **Nrtn** | mmu-let-7b | mmu-let-7d |  |  |  |
| **NRXN1** | mmu-miR-339-5p | mmu-miR-128 | |  |  |
| **NSUN4** | mmu-miR-370 | |  |  |  |
| **NUMBL** | mmu-miR-203 | mmu-let-7b |  |  |  |
| **NXF1** | mmu-miR-128 | |  |  |  |
| **NXPH1** | mmu-miR-203 | mmu-miR-139-5p | |  |  |
| **Nxph3** | mmu-miR-338 | |  |  |  |
| **ODZ3** | mmu-miR-342-3p | |  |  |  |
| **Orc5l** | mmu-miR-370 | mmu-miR-128 | |  |  |
| **Otud7** | mmu-let-7d |  |  |  |  |
| **OTX2** | mmu-miR-128 | |  |  |  |
| **Ovol1** | mmu-miR-203 | |  |  |  |
| **P2rx1** | mmu-let-7b |  |  |  |  |
| **P2RY5** | mmu-miR-128 | |  |  |  |
| **PAFAH1B1** | mmu-miR-339-5p | |  |  |  |
| **Paip2** | mmu-miR-128 | |  |  |  |
| **Pak6** | mmu-miR-339-5p | mmu-miR-128 | |  |  |
| **PAK7** | mmu-miR-320 | |  |  |  |
| **PANK1** | mmu-miR-128 | |  |  |  |
| **Papd4** | mmu-miR-203 | |  |  |  |
| **Paqr6** | mmu-miR-128 | |  |  |  |
| **PBX2** | mmu-let-7b |  |  |  |  |
| **PBX3** | mmu-miR-320 | mmu-let-7b | mmu-let-7d |  |  |
| **Pcbp2** | mmu-miR-203 | |  |  |  |
| **Pcdh1** | mmu-miR-146a | |  |  |  |
| **PCDH17** | mmu-miR-339-5p | mmu-miR-203 | |  |  |
| **PCDHA1** | mmu-miR-320 | |  |  |  |
| **PCDHA10** | mmu-miR-320 | |  |  |  |
| **PCDHA12** | mmu-miR-320 | |  |  |  |
| **PCDHA13** | mmu-miR-320 | |  |  |  |
| **PCDHA2** | mmu-miR-320 | |  |  |  |
| **PCDHA3** | mmu-miR-320 | |  |  |  |
| **PCDHA4** | mmu-miR-320 | |  |  |  |
| **PCDHA5** | mmu-miR-320 | |  |  |  |
| **PCDHA6** | mmu-miR-320 | |  |  |  |
| **PCDHA7** | mmu-miR-320 | |  |  |  |
| **PCDHA8** | mmu-miR-320 | |  |  |  |
| **PCDHAC1** | mmu-miR-320 | |  |  |  |
| **Pcf11** | mmu-miR-139-5p | |  |  |  |
| **PCNX** | mmu-miR-337 | |  |  |  |
| **PCSK2** | mmu-miR-203 | |  |  |  |
| **PCSK7** | mmu-miR-320 | |  |  |  |
| **Pctk1** | mmu-miR-370 | |  |  |  |
| **PCTK3** | mmu-miR-128 | |  |  |  |
| **PCYT1A** | mmu-miR-339-5p | |  |  |  |
| **PDAP1** | mmu-miR-203 | |  |  |  |
| **Pdcd10** | mmu-miR-203 | |  |  |  |
| **Pde3a** | mmu-miR-139-5p | mmu-miR-128 | |  |  |
| **Pde4a** | mmu-miR-139-5p | |  |  |  |
| **PDE7B** | mmu-miR-337 | mmu-miR-128 | |  |  |
| **PDGFRA** | mmu-miR-342-3p | mmu-miR-128 | |  |  |
| **PDIA5** | mmu-miR-128 | |  |  |  |
| **PDIK1L** | mmu-miR-139-5p | |  |  |  |
| **PELI3** | mmu-miR-128 | |  |  |  |
| **PER1** | mmu-miR-146a | |  |  |  |
| **Pfkfb4** | mmu-let-7b |  |  |  |  |
| **Pfkm** | mmu-miR-320 | |  |  |  |
| **PFKM** | mmu-miR-128 | |  |  |  |
| **PFN2** | mmu-miR-342-3p | |  |  |  |
| **PFTK1** | mmu-miR-339-5p | |  |  |  |
| **PGM2L1** | mmu-miR-128 | |  |  |  |
| **Phb** | mmu-miR-128 | |  |  |  |
| **Phc2** | mmu-miR-328 | |  |  |  |
| **Phf1** | mmu-miR-320 | |  |  |  |
| **Phf12** | mmu-miR-203 | |  |  |  |
| **Phf15** | mmu-miR-328 | |  |  |  |
| **PHF19** | mmu-miR-203 | |  |  |  |
| **PHF2** | mmu-miR-203 | |  |  |  |
| **PHF6** | mmu-miR-139-5p | mmu-miR-128 | |  |  |
| **Phf8** | mmu-let-7d | mmu-miR-320 | |  |  |
| **Phlda1** | mmu-miR-203 | |  |  |  |
| **PHLDA3** | mmu-miR-203 | |  |  |  |
| **PHOX2B** | mmu-miR-146a | mmu-miR-320 | |  |  |
| **PIB5PA** | mmu-miR-128 | |  |  |  |
| **Piga** | mmu-let-7d |  |  |  |  |
| **Pik3ca** | mmu-miR-320 | |  |  |  |
| **PIM1** | mmu-miR-328 | |  |  |  |
| **PIM2** | mmu-miR-339-5p | |  |  |  |
| **Pitx2** | mmu-miR-328 | mmu-miR-339-5p | mmu-miR-203 | |  |
| **PKIA** | mmu-miR-128 | |  |  |  |
| **Pkp3** | mmu-miR-339-5p | |  |  |  |
| **PLAG1** | mmu-miR-128 | |  |  |  |
| **PLAGL2** | mmu-let-7b | mmu-miR-128 | |  |  |
| **Plcd1** | mmu-miR-191 | |  |  |  |
| **Pld2** | mmu-miR-203 | |  |  |  |
| **PLD3** | mmu-miR-338 | |  |  |  |
| **Pldn** | mmu-let-7d |  |  |  |  |
| **PLEKHH2** | mmu-miR-128 | |  |  |  |
| **PLK2** | mmu-miR-342-3p | mmu-miR-320 | mmu-miR-128 | |  |
| **Plk3** | mmu-miR-320 | |  |  |  |
| **PLXND1** | mmu-miR-128 | |  |  |  |
| **PODXL** | mmu-miR-339-5p | |  |  |  |
| **POGZ** | mmu-let-7b |  |  |  |  |
| **POM121** | mmu-miR-342-3p | |  |  |  |
| **POMT2** | mmu-miR-339-5p | |  |  |  |
| **POU3F2** | mmu-miR-128 | |  |  |  |
| **PPAP2B** | mmu-miR-203 | |  |  |  |
| **PPAPDC2** | mmu-let-7b |  |  |  |  |
| **Pparbp** | mmu-miR-146a | |  |  |  |
| **Pparg** | mmu-miR-128 | |  |  |  |
| **Ppargc1a** | mmu-miR-320 | |  |  |  |
| **Ppm1b** | mmu-miR-320 | |  |  |  |
| **PPM1D** | mmu-miR-203 | |  |  |  |
| **PPM1E** | mmu-miR-203 | mmu-miR-128 | |  |  |
| **PPM1F** | mmu-miR-342-3p | |  |  |  |
| **PPP1CB** | mmu-miR-203 | |  |  |  |
| **PPP1CC** | mmu-miR-128 | |  |  |  |
| **Ppp1r3f** | mmu-miR-342-3p | |  |  |  |
| **PPP2CA** | mmu-miR-139-5p | |  |  |  |
| **PPP2R1B** | mmu-miR-337 | |  |  |  |
| **PPP2R2C** | mmu-miR-320 | |  |  |  |
| **Ppp2r5b** | mmu-miR-320 | |  |  |  |
| **PPP4C** | mmu-miR-128 | |  |  |  |
| **Ppp4r1** | mmu-miR-338 | |  |  |  |
| **Ppp6c** | mmu-let-7d |  |  |  |  |
| **Prdm10** | mmu-miR-203 | |  |  |  |
| **Prdm15** | mmu-miR-139-5p | |  |  |  |
| **PRICKLE2** | mmu-miR-128 | |  |  |  |
| **PRKAG2** | mmu-miR-203 | |  |  |  |
| **PRKCA** | mmu-miR-203 | |  |  |  |
| **PRKCB1** | mmu-miR-203 | |  |  |  |
| **PRKD1** | mmu-miR-128 | |  |  |  |
| **PRKG1** | mmu-miR-203 | |  |  |  |
| **ProSAPiP1** | mmu-miR-320 | mmu-miR-128 | |  |  |
| **PRPF19** | mmu-miR-203 | |  |  |  |
| **Prpf38b** | mmu-let-7b | mmu-let-7d |  |  |  |
| **PRPF4B** | mmu-miR-139-5p | mmu-miR-146a | |  |  |
| **PRPS2** | mmu-miR-203 | |  |  |  |
| **Prrx2** | mmu-miR-320 | |  |  |  |
| **PRX** | mmu-miR-146a | |  |  |  |
| **PSCD1** | mmu-miR-339-5p | |  |  |  |
| **PSMA1** | mmu-miR-128 | |  |  |  |
| **Psmd3** | mmu-miR-338 | mmu-miR-146a | mmu-let-7d |  |  |
| **PSME3** | mmu-miR-328 | |  |  |  |
| **Psors1c2** | mmu-let-7d | mmu-let-7b |  |  |  |
| **Pspc1** | mmu-miR-203 | |  |  |  |
| **PTEN** | mmu-miR-337 | |  |  |  |
| **PTER** | mmu-miR-128 | |  |  |  |
| **PTGER4** | mmu-miR-342-3p | mmu-miR-128 | |  |  |
| **PTGFRN** | mmu-miR-146a | |  |  |  |
| **Ptk7** | mmu-miR-328 | |  |  |  |
| **Ptp4a1** | mmu-miR-339-5p | |  |  |  |
| **PTPN12** | mmu-miR-338 | |  |  |  |
| **PTPN3** | mmu-miR-128 | |  |  |  |
| **PTPN4** | mmu-miR-128 | |  |  |  |
| **PTPN5** | mmu-miR-128 | |  |  |  |
| **Ptpn9** | mmu-miR-328 | mmu-miR-128 | |  |  |
| **Ptprn** | mmu-miR-342-3p | |  |  |  |
| **PTPRT** | mmu-miR-128 | |  |  |  |
| **Ptpru** | mmu-miR-139-5p | |  |  |  |
| **PTRF** | mmu-miR-370 | |  |  |  |
| **Qars** | mmu-let-7d | mmu-let-7b |  |  |  |
| **QKI** | mmu-miR-128 | |  |  |  |
| **RAB10** | mmu-miR-203 | |  |  |  |
| **RAB11FIP1** | mmu-miR-128 | |  |  |  |
| **Rab11fip4** | mmu-let-7d |  |  |  |  |
| **RAB14** | mmu-miR-338 | mmu-miR-320 | |  |  |
| **RAB15** | mmu-let-7b |  |  |  |  |
| **RAB18** | mmu-miR-320 | |  |  |  |
| **RAB1A** |  |  |  |  |  |
| **Rab3ip** | mmu-miR-342-3p | |  |  |  |
| **RAB8B** | mmu-miR-203 | |  |  |  |
| **Rad18** | mmu-miR-320 | |  |  |  |
| **Rai2** | mmu-miR-320 | |  |  |  |
| **RALA** |  |  |  |  |  |
| **RAP1A** | mmu-miR-203 | mmu-miR-320 | |  |  |
| **RAP1B** | mmu-miR-339-5p | mmu-miR-139-5p | mmu-miR-128 | |  |
| **RAP1GDS1** | mmu-miR-370 | |  |  |  |
| **RAP2A** | mmu-miR-203 | |  |  |  |
| **RAP2C** | mmu-miR-320 | |  |  |  |
| **RARA** | mmu-miR-128 | |  |  |  |
| **RARB** | mmu-miR-203 | mmu-miR-146a | |  |  |
| **Rasa1** | mmu-miR-342-3p | |  |  |  |
| **RASGEF1B** | mmu-miR-128 | |  |  |  |
| **Rasgrp2** | mmu-miR-328 | |  |  |  |
| **RASL10B** | mmu-miR-337 | mmu-let-7d |  |  |  |
| **Rassf1** | mmu-miR-339-5p | mmu-miR-342-3p | |  |  |
| **RBPMS2** | mmu-miR-342-3p | |  |  |  |
| **Rdh10** | mmu-let-7d |  |  |  |  |
| **RDX** | mmu-miR-337 | |  |  |  |
| **RELN** | mmu-miR-128 | |  |  |  |
| **REPS1** | mmu-miR-128 | |  |  |  |
| **REPS2** | mmu-miR-337 | |  |  |  |
| **RERE** | mmu-miR-128 | |  |  |  |
| **Rev1l** | mmu-miR-203 | |  |  |  |
| **REV3L** | mmu-miR-203 | |  |  |  |
| **Rfxdc1** | mmu-let-7b | mmu-let-7d |  |  |  |
| **Rfxdc2** | mmu-miR-370 | |  |  |  |
| **RGAG4** | mmu-miR-370 | |  |  |  |
| **Rgl2** | mmu-miR-128 | |  |  |  |
| **Rgnef** | mmu-let-7d |  |  |  |  |
| **RGS1** | mmu-miR-128 | |  |  |  |
| **RGS6** | mmu-miR-128 | |  |  |  |
| **Rhcg** | mmu-miR-370 | |  |  |  |
| **RHOG** | mmu-miR-320 | |  |  |  |
| **RIMS3** | mmu-miR-128 | |  |  |  |
| **RIN2** | mmu-miR-338 | |  |  |  |
| **RNF111** | mmu-miR-337 | mmu-miR-203 | |  |  |
| **RNF139** | mmu-miR-191 | |  |  |  |
| **RNF139** | mmu-miR-128 | |  |  |  |
| **RNF144** | mmu-miR-128 | |  |  |  |
| **Rnf20** | mmu-let-7b |  |  |  |  |
| **Rnf25** | mmu-miR-339-5p | |  |  |  |
| **Rnf34** | mmu-miR-203 | mmu-miR-320 | |  |  |
| **RNF38** | mmu-miR-203 | |  |  |  |
| **RNF38** | mmu-miR-128 | |  |  |  |
| **RNF4** | mmu-miR-146a | |  |  |  |
| **Rnf5** | mmu-let-7b | mmu-let-7d |  |  |  |
| **Rnf7** | mmu-let-7d |  |  |  |  |
| **RNGTT** | mmu-miR-128 | |  |  |  |
| **ROBO1** | mmu-miR-146a | |  |  |  |
| **Robo2** | mmu-let-7b |  |  |  |  |
| **RORB** | mmu-miR-128 | |  |  |  |
| **RPS19** | mmu-miR-338 | |  |  |  |
| **RPS6KA5** | mmu-miR-128 | |  |  |  |
| **RPS6KB1** | mmu-miR-320 | |  |  |  |
| **RRM2** | mmu-let-7d |  |  |  |  |
| **Rsbn1** | mmu-miR-342-3p | mmu-miR-128 | |  |  |
| **RSPO2** | mmu-let-7d |  |  |  |  |
| **RUFY3** | mmu-miR-139-5p | mmu-let-7d | mmu-let-7b |  |  |
| **RUNX1** | mmu-miR-320 | mmu-miR-128 | |  |  |
| **RUNX2** | mmu-miR-338 | mmu-miR-203 | |  |  |
| **Rusc2** | mmu-miR-338 | |  |  |  |
| **RXRA** | mmu-miR-128 | |  |  |  |
| **RYBP** | mmu-miR-337 | mmu-miR-128 | |  |  |
| **SAMD10** | mmu-miR-128 | |  |  |  |
| **SASH1** | mmu-miR-128 | |  |  |  |
| **SATB2** | mmu-miR-128 | |  |  |  |
| **Schip1** | mmu-miR-139-5p | |  |  |  |
| **Scn11a** | mmu-let-7b |  |  |  |  |
| **Scotin** | mmu-miR-328 | |  |  |  |
| **SDHC** | mmu-miR-320 | |  |  |  |
| **Sec24a** | mmu-miR-128 | |  |  |  |
| **SEC24D** | mmu-miR-203 | |  |  |  |
| **Sec61a1** | mmu-miR-128 | |  |  |  |
| **SEMA3A** | mmu-miR-320 | |  |  |  |
| **SEMA3D** | mmu-miR-342-3p | |  |  |  |
| **SEMA3G** | mmu-miR-146a | mmu-miR-203 | |  |  |
| **SEMA4F** | mmu-miR-338 | |  |  |  |
| **Sema4g** | mmu-miR-203 | mmu-miR-342-3p | |  |  |
| **SEMA5A** | mmu-miR-203 | |  |  |  |
| **SEMA6A** | mmu-miR-128 | |  |  |  |
| **SEMA6D** | mmu-miR-320 | |  |  |  |
| **SENP1** | mmu-miR-339-5p | |  |  |  |
| **SENP5** | mmu-let-7b |  |  |  |  |
| **SERBP1** | mmu-miR-320 | |  |  |  |
| **SERTAD2** | mmu-miR-328 | |  |  |  |
| **SESTD1** | mmu-miR-203 | |  |  |  |
| **SET** | mmu-miR-338 | mmu-miR-146a | |  |  |
| **Sf3b4** |  |  |  |  |  |
| **SFRP1** | mmu-miR-128 | |  |  |  |
| **SFRS1** | mmu-miR-139-5p | mmu-miR-146a | mmu-miR-203 | |  |
| **Sfrs11** | mmu-miR-370 | |  |  |  |
| **SFRS12** | mmu-miR-337 | mmu-miR-128 | |  |  |
| **SFRS2** | mmu-miR-338 | |  |  |  |
| **Sfrs4** | mmu-miR-139-5p | |  |  |  |
| **SFRS6** | mmu-miR-146a | |  |  |  |
| **Sfrs7** | mmu-miR-320 | |  |  |  |
| **Sfrs8** | mmu-miR-320 | mmu-miR-370 | |  |  |
| **Sgms1** | mmu-miR-128 | |  |  |  |
| **SGPP1** | mmu-miR-128 | |  |  |  |
| **Sgsh** | mmu-miR-370 | |  |  |  |
| **Sh2d3c** | mmu-miR-128 | |  |  |  |
| **SH3BGRL2** | mmu-miR-128 | mmu-miR-146a | |  |  |
| **SH3RF1** | mmu-miR-128 | |  |  |  |
| **SHC1** | mmu-miR-203 | |  |  |  |
| **SHOC2** | mmu-miR-128 | |  |  |  |
| **SIRT1** | mmu-miR-128 | |  |  |  |
| **SIX4** | mmu-miR-328 | |  |  |  |
| **Slamf6** | mmu-miR-370 | |  |  |  |
| **SLC10A3** | mmu-miR-320 | |  |  |  |
| **SLC12A2** | mmu-miR-203 | |  |  |  |
| **Slc12a9** | mmu-let-7b | mmu-let-7d |  |  |  |
| **SLC17A5** | mmu-miR-337 | |  |  |  |
| **SLC17A6** | mmu-miR-203 | |  |  |  |
| **Slc1a2** | mmu-miR-338 | mmu-miR-128 | |  |  |
| **SLC20A1** | mmu-miR-339-5p | mmu-let-7d |  |  |  |
| **SLC23A2** | mmu-miR-139-5p | mmu-miR-203 | |  |  |
| **Slc25a14** | mmu-miR-146a | |  |  |  |
| **SLC25A27** | mmu-let-7d |  |  |  |  |
| **Slc25a3** | mmu-miR-139-5p | |  |  |  |
| **Slc2a1** | mmu-miR-328 | |  |  |  |
| **SLC2A10** | mmu-miR-338 | |  |  |  |
| **SLC30A7** | mmu-miR-338 | |  |  |  |
| **Slc38a3** | mmu-miR-370 | |  |  |  |
| **SLC38A4** | mmu-miR-128 | |  |  |  |
| **SLC39A10** | mmu-miR-203 | |  |  |  |
| **SLC39A11** | mmu-miR-128 | |  |  |  |
| **SLC39A13** | mmu-miR-128 | |  |  |  |
| **Slc39a5** | mmu-miR-328 | |  |  |  |
| **SLC4A10** | mmu-miR-339-5p | |  |  |  |
| **SLC6A1** | mmu-miR-128 | |  |  |  |
| **SLC6A6** | mmu-miR-203 | |  |  |  |
| **Slc7a10** | mmu-miR-128 | |  |  |  |
| **SLC7A11** | mmu-miR-128 | |  |  |  |
| **SLC7A14** | mmu-miR-203 | |  |  |  |
| **Slc8a2** | mmu-let-7b |  |  |  |  |
| **SLCO5A1** | mmu-miR-128 | |  |  |  |
| **SLITRK1** | mmu-miR-128 | |  |  |  |
| **Slitrk3** | mmu-miR-328 | |  |  |  |
| **Slmap** | mmu-miR-338 | |  |  |  |
| **SMAP1** | mmu-miR-320 | mmu-miR-339-5p | |  |  |
| **SMARCA1** | mmu-miR-128 | |  |  |  |
| **Smarca2** | mmu-miR-320 | |  |  |  |
| **Smarca4** | mmu-miR-139-5p | |  |  |  |
| **SMG1** | mmu-miR-128 | |  |  |  |
| **SMOX** | mmu-miR-139-5p | |  |  |  |
| **Smtn** | mmu-miR-338 | |  |  |  |
| **SMURF1** | mmu-miR-203 | |  |  |  |
| **SNAP25** | mmu-miR-342-3p | mmu-miR-128 | |  |  |
| **SNF1LK** | mmu-miR-203 | |  |  |  |
| **Snf8** | mmu-miR-370 | |  |  |  |
| **SNRK** | mmu-miR-328 | |  |  |  |
| **SNX16** | mmu-let-7b | mmu-let-7d |  |  |  |
| **SNX4** | mmu-miR-338 | |  |  |  |
| **SOCS3** | mmu-miR-203 | |  |  |  |
| **SOCS6** | mmu-miR-203 | mmu-miR-128 | |  |  |
| **Son** | mmu-miR-338 | mmu-miR-320 | |  |  |
| **SORCS3** | mmu-miR-342-3p | |  |  |  |
| **Sostdc1** | mmu-miR-203 | |  |  |  |
| **SOX11** | mmu-miR-328 | |  |  |  |
| **SOX13** | mmu-miR-203 | |  |  |  |
| **Sox30** | mmu-miR-203 | |  |  |  |
| **SOX6** | mmu-miR-203 | mmu-miR-342-3p | |  |  |
| **SP1** | mmu-miR-128 | |  |  |  |
| **SP2** | mmu-miR-128 | |  |  |  |
| **SP4** | mmu-miR-337 | mmu-miR-203 | |  |  |
| **SP6** | mmu-miR-338 | |  |  |  |
| **SPATA2** | mmu-miR-128 | |  |  |  |
| **Spcs2** | mmu-miR-320 | |  |  |  |
| **Spen** | mmu-miR-203 | |  |  |  |
| **SPIRE1** | mmu-miR-203 | |  |  |  |
| **SPRY2** | mmu-miR-128 | |  |  |  |
| **Srp19** | mmu-miR-320 | |  |  |  |
| **SRR** | mmu-miR-146a | |  |  |  |
| **SS18L1** | mmu-miR-128 | |  |  |  |
| **Ssb** | mmu-miR-338 | |  |  |  |
| **Ssx2ip** | mmu-miR-139-5p | |  |  |  |
| **ST14** | mmu-miR-128 | |  |  |  |
| **St3gal3** | mmu-miR-328 | |  |  |  |
| **STAG1** | mmu-miR-128 | |  |  |  |
| **STAG2** | mmu-miR-338 | |  |  |  |
| **Stard3nl** | mmu-miR-203 | |  |  |  |
| **STC1** | mmu-miR-146a | |  |  |  |
| **Steap1** | mmu-miR-203 | |  |  |  |
| **STEAP3** | mmu-miR-128 | |  |  |  |
| **STIM2** | mmu-miR-128 | |  |  |  |
| **STK24** | mmu-miR-128 | |  |  |  |
| **STK35** | mmu-miR-128 | |  |  |  |
| **STK39** | mmu-miR-128 | |  |  |  |
| **Strbp** | mmu-miR-146a | |  |  |  |
| **STRN4** | mmu-miR-128 | |  |  |  |
| **STX16** | mmu-miR-128 | |  |  |  |
| **STX17** | mmu-let-7d |  |  |  |  |
| **STX3** | mmu-miR-146a | |  |  |  |
| **SUHW2** | mmu-let-7d |  |  |  |  |
| **Suhw3** | mmu-miR-320 | |  |  |  |
| **SV2A** | mmu-miR-128 | |  |  |  |
| **SYDE1** | mmu-miR-128 | |  |  |  |
| **Syn2** | mmu-miR-139-5p | |  |  |  |
| **SYT1** | mmu-miR-146a | mmu-miR-128 | |  |  |
| **SYT4** | mmu-miR-128 | |  |  |  |
| **Syt6** | mmu-miR-320 | |  |  |  |
| **Taf12** | mmu-miR-337 | |  |  |  |
| **TAF4** | mmu-miR-128 | |  |  |  |
| **TAF5** | mmu-miR-337 | mmu-miR-191 | mmu-miR-203 | mmu-miR-320 | |
| **TAF9B** | mmu-miR-146a | |  |  |  |
| **TANC1** | mmu-miR-338 | |  |  |  |
| **Taok1** | mmu-miR-370 | |  |  |  |
| **Tbc1d15** | mmu-miR-338 | |  |  |  |
| **TBC1D22B** | mmu-miR-128 | |  |  |  |
| **Tbkbp1** | mmu-miR-328 | |  |  |  |
| **Tbx1** | mmu-miR-139-5p | |  |  |  |
| **TBX5** | mmu-miR-128 | |  |  |  |
| **TCF20** | mmu-miR-128 | |  |  |  |
| **TDG** | mmu-miR-320 | |  |  |  |
| **TEAD3** | mmu-let-7d |  |  |  |  |
| **TESK2** | mmu-miR-328 | |  |  |  |
| **TFAP4** | mmu-miR-339-5p | |  |  |  |
| **Tfdp2** | mmu-miR-342-3p | |  |  |  |
| **TFEB** | mmu-miR-128 | |  |  |  |
| **TGFB3** | mmu-miR-203 | |  |  |  |
| **THRA** | mmu-miR-339-5p | |  |  |  |
| **THRAP1** | mmu-miR-128 | |  |  |  |
| **THRAP2** | mmu-miR-128 | |  |  |  |
| **TIA1** | mmu-miR-339-5p | |  |  |  |
| **TIAM1** | mmu-miR-337 | |  |  |  |
| **Tjp1** | mmu-miR-191 | |  |  |  |
| **TLK1** | mmu-miR-203 | |  |  |  |
| **TLK2** | mmu-miR-320 | mmu-miR-128 | |  |  |
| **TLOC1** | mmu-miR-203 | |  |  |  |
| **TMCC1** | mmu-miR-128 | |  |  |  |
| **TMED5** | mmu-miR-128 | |  |  |  |
| **TMEFF1** | mmu-miR-128 | |  |  |  |
| **TMEM100** | mmu-miR-203 | |  |  |  |
| **Tmem108** | mmu-miR-320 | |  |  |  |
| **TMEM110** | mmu-miR-128 | |  |  |  |
| **TMEM2** | mmu-miR-320 | mmu-let-7b |  |  |  |
| **Tmem23** | mmu-miR-203 | |  |  |  |
| **TMEM25** | mmu-miR-128 | |  |  |  |
| **TMEM28** | mmu-miR-128 | |  |  |  |
| **Tmem35** | mmu-miR-342-3p | |  |  |  |
| **Tmem41a** | mmu-let-7d |  |  |  |  |
| **TMEM47** | mmu-miR-320 | |  |  |  |
| **Tmem58** | mmu-miR-337 | |  |  |  |
| **TMEM64** | mmu-miR-320 | |  |  |  |
| **TMEM9B** | mmu-miR-320 | mmu-miR-128 | |  |  |
| **Tmod2** | mmu-miR-191 | |  |  |  |
| **Tmsb10** | mmu-miR-128 | |  |  |  |
| **TMTC2** | mmu-miR-342-3p | mmu-miR-128 | |  |  |
| **TNFRSF19** | mmu-miR-337 | |  |  |  |
| **TNFRSF1B** | mmu-let-7b |  |  |  |  |
| **Tnfrsf21** | mmu-miR-320 | |  |  |  |
| **TNPO3** | mmu-miR-128 | |  |  |  |
| **TNRC6B** | mmu-miR-337 | mmu-miR-338 | mmu-miR-203 | |  |
| **TOB2** | mmu-let-7d |  |  |  |  |
| **TOMM70A** | mmu-miR-320 | |  |  |  |
| **Top1** | mmu-miR-337 | |  |  |  |
| **TPD52** | mmu-miR-139-5p | |  |  |  |
| **TPD52L2** | mmu-miR-337 | |  |  |  |
| **Tpm3** | mmu-miR-338 | |  |  |  |
| **Traf6** | mmu-miR-146a | |  |  |  |
| **Traf7** | mmu-miR-320 | |  |  |  |
| **Traip** | mmu-miR-370 | |  |  |  |
| **Trap1** | mmu-miR-320 | |  |  |  |
| **TRIM2** | mmu-miR-338 | |  |  |  |
| **Trim41** | mmu-miR-320 | |  |  |  |
| **TROVE2** | mmu-miR-139-5p | mmu-miR-320 | mmu-miR-128 | |  |
| **Trp73** | mmu-miR-128 | |  |  |  |
| **Trpc3** | mmu-miR-339-5p | |  |  |  |
| **Trpc7** | mmu-miR-139-5p | |  |  |  |
| **TSC1** | mmu-let-7d |  |  |  |  |
| **TSC22D1** | mmu-miR-203 | |  |  |  |
| **Tsc22d2** | mmu-miR-337 | mmu-miR-203 | |  |  |
| **Tsc22d3** | mmu-miR-320 | |  |  |  |
| **Tsc22d4** | mmu-miR-320 | |  |  |  |
| **Tspan18** | mmu-let-7b | mmu-let-7d |  |  |  |
| **Tspan3** | mmu-miR-139-5p | |  |  |  |
| **TSPAN5** | mmu-miR-139-5p | |  |  |  |
| **TSR1** | mmu-miR-338 | |  |  |  |
| **Ttc9c** | mmu-let-7d |  |  |  |  |
| **Ttll4** | mmu-let-7b | mmu-let-7d |  |  |  |
| **TUSC2** | mmu-let-7b | mmu-let-7d | mmu-miR-203 | |  |
| **TWF1** | mmu-miR-203 | |  |  |  |
| **UBB** | mmu-miR-337 | |  |  |  |
| **UBE2D3** | mmu-miR-320 | |  |  |  |
| **UBE2E2** | mmu-miR-128 | |  |  |  |
| **Ube2e3** | mmu-miR-139-5p | mmu-miR-128 | |  |  |
| **UBE2F** | mmu-miR-128 | |  |  |  |
| **Ube2g1** | mmu-miR-338 | |  |  |  |
| **UBE2N** | mmu-miR-128 | |  |  |  |
| **UBE2Q1** | mmu-miR-128 | |  |  |  |
| [**UBE2V1**](http://www.ncbi.nlm.nih.gov/sites/entrez?Db=gene&Cmd=ShowDetailView&TermToSearch=7335) | mmu-miR-128 | |  |  |  |
| **UBE2W** | mmu-miR-128 | |  |  |  |
| **UBE2Z** | mmu-miR-328 | mmu-miR-128 | |  |  |
| **UBE4A** | mmu-miR-128 | |  |  |  |
| **UBLCP1** | mmu-miR-338 | |  |  |  |
| **Ubn1** | mmu-miR-342-3p | |  |  |  |
| **Ubr1** | mmu-miR-203 | |  |  |  |
| **Ulk1** | mmu-miR-320 | |  |  |  |
| **ULK2** | mmu-miR-328 | |  |  |  |
| **UNC45B** | mmu-miR-338 | |  |  |  |
| **UNC50** | mmu-miR-203 | |  |  |  |
| **UNC5A** | mmu-let-7d | mmu-miR-203 | mmu-miR-320 | |  |
| **UNC5D** | mmu-miR-128 | |  |  |  |
| **Usf2** | mmu-miR-339-5p | |  |  |  |
| **Usp20** | mmu-miR-320 | |  |  |  |
| **Usp21** | mmu-let-7b | mmu-let-7d |  |  |  |
| **Usp3** | mmu-miR-146a | mmu-miR-203 | |  |  |
| **Usp37** | mmu-miR-328 | |  |  |  |
| **Usp42** | mmu-miR-128 | |  |  |  |
| **UXS1** | mmu-miR-337 | |  |  |  |
| **VAMP2** | mmu-miR-338 | |  |  |  |
| **VAPA** | mmu-miR-203 | |  |  |  |
| **Vat1** | mmu-miR-339-5p | |  |  |  |
| **VAV3** | mmu-miR-203 | mmu-miR-128 | |  |  |
| **Vcp** | mmu-miR-339-5p | |  |  |  |
| **VEGFA** | mmu-miR-342-3p | |  |  |  |
| **VEGFB** | mmu-miR-128 | |  |  |  |
| **VIP** | mmu-miR-128 | |  |  |  |
| **Vps37b** | mmu-miR-320 | |  |  |  |
| **VPS4B** | mmu-miR-128 | |  |  |  |
| **Vsig4** | mmu-miR-328 | |  |  |  |
| **Wasl** | mmu-let-7b |  |  |  |  |
| **Wdr40a** | mmu-miR-146a | |  |  |  |
| **Wdr42a** | mmu-let-7d |  |  |  |  |
| **WDR47** | mmu-miR-139-5p | |  |  |  |
| **Wdr6** | mmu-miR-337 | |  |  |  |
| **WDR68** | mmu-miR-128 | |  |  |  |
| **Wdr77** | mmu-miR-342-3p | |  |  |  |
| **Wdr81** | mmu-miR-339-5p | |  |  |  |
| [**WDTC1**](http://www.ncbi.nlm.nih.gov/sites/entrez?Db=gene&Cmd=ShowDetailView&TermToSearch=23038) | mmu-miR-128 | |  |  |  |
| **WEE1** | mmu-miR-128 | |  |  |  |
| **Wnt10b** | mmu-miR-370 | |  |  |  |
| **WNT3A** | mmu-miR-128 | |  |  |  |
| **WRNIP1** | mmu-miR-320 | |  |  |  |
| **WSB1** | mmu-miR-128 | |  |  |  |
| **WTAP** | mmu-miR-139-5p | mmu-miR-203 | mmu-miR-128 | |  |
| **XBP1** | mmu-miR-320 | |  |  |  |
| **XKR4** | mmu-miR-370 | |  |  |  |
| **XPO1** | mmu-miR-320 | |  |  |  |
| **XPR1** | mmu-miR-128 | |  |  |  |
| **Xrn2** | mmu-miR-203 | |  |  |  |
| **Ypel3** | mmu-miR-128 | |  |  |  |
| **Yrdc** | mmu-miR-338 | |  |  |  |
| **YTHDF3** | mmu-miR-203 | mmu-miR-320 | |  |  |
| **YWHAB** | mmu-miR-337 | mmu-miR-128 | |  |  |
| **Ywhae** | mmu-miR-320 | |  |  |  |
| **YWHAG** | mmu-miR-139-5p | |  |  |  |
| **YWHAH** | mmu-miR-338 | |  |  |  |
| **YWHAQ** | mmu-miR-139-5p | mmu-miR-203 | mmu-miR-320 | |  |
| **ZADH2** | mmu-miR-128 | |  |  |  |
| **Zbtb10** | mmu-miR-338 | |  |  |  |
| **Zbtb20** | mmu-miR-203 | |  |  |  |
| **ZBTB39** | mmu-miR-370 | |  |  |  |
| **Zc3h3** | mmu-let-7b |  |  |  |  |
| **ZC3H7A** | mmu-miR-338 | |  |  |  |
| **Zcchc11** | mmu-let-7b |  |  |  |  |
| **Zdhhc17** | mmu-miR-146a | mmu-miR-128 | |  |  |
| **Zdhhc18** | mmu-miR-338 | |  |  |  |
| **ZDHHC21** | mmu-miR-338 | |  |  |  |
| **ZFAND3** | mmu-miR-338 | mmu-miR-139-5p | mmu-miR-181a-1* | |  |
| **ZFHX4** | mmu-miR-338 | mmu-miR-203 | mmu-miR-128 | |  |
| **Zfp148** | mmu-miR-370 | |  |  |  |
| **Zfp238** | mmu-miR-338 | |  |  |  |
| **Zfp281** | mmu-miR-320 | |  |  |  |
| **Zfp282** | mmu-let-7b |  |  |  |  |
| **Zfp354a** | mmu-let-7d |  |  |  |  |
| **Zfp367** | mmu-miR-146a | |  |  |  |
| **ZFP36L1** | mmu-miR-139-5p | |  |  |  |
| **Zfp462** | mmu-miR-342-3p | |  |  |  |
| **Zfp622** | mmu-miR-320 | |  |  |  |
| **ZFX** | mmu-miR-342-3p | mmu-miR-139-5p | mmu-miR-146a | |  |
| **ZFYVE1** | mmu-miR-146a | |  |  |  |
| **ZHX1** | mmu-miR-128 | |  |  |  |
| **ZIC3** | mmu-miR-320 | |  |  |  |
| **ZIC4** | mmu-miR-337 | mmu-miR-342-3p | |  |  |
| **Zmynd11** | mmu-miR-370 | mmu-miR-203 | |  |  |
| **ZNF192** | mmu-miR-128 | |  |  |  |
| **ZNF385** | mmu-miR-128 | |  |  |  |
| **ZNRF2** | mmu-miR-339-5p | |  |  |  |
| **ZZZ3** | mmu-miR-338 | mmu-miR-342-3p | mmu-let-7b | mmu-miR-128 | |
